# Computational modelling reveals novel insights into GnRH receptor activation and binding dynamics

**DOI:** 10.1038/s41598-025-26518-8

**Published:** 2025-11-27

**Authors:** Elpiniki Paspali, Valerie Anne Ferro, Karina Kubiak-Ossowska, Paul Alexander Mulheran

**Affiliations:** 1 Chemical & Process Engineering, University of Strathclyde, G1 1XL Glasgow, UK; 2 Strathclyde Institute of Pharmacy & Biomedical Sciences, University of Strathclyde, G4 0RE Glasgow, UK; 3 Department of Physics/ARCHIE-WeSt, University of Strathclyde, G4 0NG Glasgow, UK

**Keywords:** Computational models, Protein function predictions, Protein structure predictions, Computational chemistry, Chemical biology, Membranes, Gonadal hormones, G protein-coupled receptors

## Abstract

Gonadotrophin-releasing hormone (GnRH) regulates the mammalian reproductive system by binding to its receptor (GnRH1R) and is a target for treating reproductive hormone-dependent disorders and cancers. While the inactive structure of GnRH1R is known, the active conformation and GnRH binding mode that lead to receptor activation are not fully understood. The mechanism of GnRH-induced receptor activation remains poorly understood due to the absence of experimental structures of the active GnRH1R-GnRH complex. To address this gap, we employed computational docking simulations using Rosetta, coupled with a custom Python-based elimination protocol, to identify near-native binding poses. This approach yielded two top-ranked candidates, ROS-1 and ROS-2. Molecular dynamics simulations revealed that ROS-1 induced GnRH1R activation within 1.0 $\mu$s, characterised by a $\approx$ 4 Å outward shift of the cytoplasmic end of TM6. Key interactions included $\pi -\pi$ stacking between GnRH and GnRH1R (notably Y5 with Y283$\phantom{0}^{6.51}$, Y290$\phantom{0}^{6.58}$, and F309$\phantom{0}^{7.38}$) and hydrogen bonds with L286$\phantom{0}^{6.54}$. Intramolecular $\pi -\pi$ interactions within GnRH (Y5 and W3) also played a significant role. Two main communication pathways initiated by R8 of GnRH were identified. R8 formed cation-$\pi$ interactions with W280$\phantom{0}^{6.48}$ and communicated with N87$\phantom{0}^{2.50}$ and the DPxxY motif *via* water-mediated hydrogen bonds. Additional interactions involved M125$\phantom{0}^{3.36}$ and the PAF and DRS motifs, which are critical for receptor activation. Key differences in $\pi -\pi$ interactions at they cytosolic end of TM7 between active and inactive states were identified due to the reorganisation of the DPxxY motif. Finally, GnRH1R communication with lipids through hydrogen bonds involving R240$\phantom{0}^{5.67}$, R75$\phantom{0}^{2.38}$, and S140$\phantom{0}^{3.51}$ was observed. This study provides insights into the active conformation and binding dynamics of the GnRH-GnRH1R complex, advancing our current understanding by providing a coherent picture that consolidates previous interpretations, thereby paving the way to better therapeutic applications.

## Introduction

GnRH1R is a Class A G Protein-coupled receptor (GPCR) whose primary function is the regulation of the reproductive system, where it catalyses the synthesis of luteinising hormone (LH) and follicle-stimulating hormone (FSH) within the pituitary gland^1^. This process commences upon the binding of the GnRH hormone which is released from GnRH neurons in a pulsative manner^2–5^. Expression of GnRH1R predominantly localises at the surface of gonadotrophic cells within the pituitary gland. Hence GnRH1R is a primary target for antifertility drugs. Furthermore, GnRH1R overexpression is observed in cancers that affect gonadal steroid-dependant organs, including 86% of prostate cancers, 80% of endometrial and ovarian adenocarcinomas, and 50% of breast cancer cases^6–10^. For this reason, treatment in these cases includes direct GnRH1R inhibition. Understanding how GnRH binds to GnRH1R is therefore crucial, since it will enable the development of new drugs for a range of important applications.

The inactive conformation of the GnRH1R has been determined in complex with the antagonist elagonix^11^, however, characteristics of the active GnRH1R conformation are currently lacking. In contrast to most Class A GPCRs, the GnRH1R exhibits several variations in conserved GPCR motifs and residues (Table 1). The most prominent difference is the lack of the cytosolic C-terminal (H8) helix. In addition, the highly conserved DRY motif is replaced by the DRS motif and the conserved NPxxY motif is replaced by the charged DPxxY in GnRH1R. This difference results in a negatively charged motif in one of the transmembrane helices (TM7) that could potentially serve as a sodium-binding site^12–15^.

In order to develop more effective agonists and antagonists, it is crucial to understand in detail the binding mode of GnRH to its receptor and its subsequent activation. Prior mutagenesis studies have revealed several GnRH1R residues that either affect GnRH binding or receptor fuction^11,16–22^. Despite these extensive prior efforts, the precise binding mode of GnRH to its receptor and the conformational changes associated with receptor activation remain elusive.

In recent years, the development of in-silico methods has significantly advanced the structural characterisation of GPCRs, including binding site prediction, ligand pose sampling, and activation mechanism modelling. Several studies have demonstrated the utility of computational techniques such as molecular docking, machine learning-driven prediction models, and extensive molecular dynamics simulations in the analysis of receptor-ligand interactions and GPCR activation pathways^23–26^. Incorporating such in-silico tools not only enhances drug discovery efforts but also complements experimental limitations by providing atomistic insights into receptor dynamics and ligand specificity.

The crystal structure of the GnRH1R^11^ has provided valuable insights into the inactive state of the receptor; however, capturing the transient active state and the associated ligand-binding pose has not been achieved before. Here, we bridge the gap in our understanding of the GnRH1R activation process and GnRH binding mode by leveraging computational approaches. Specifically, we employ unbiased computational docking simulations using Rosetta https://rosettacommons.org to generate a pool of candidates and developed a Python-based protocol to select the plausible ’close to native’ GnRH-GnRH1R binding mode based on several criteria (see Methods). Furthermore, we conducted molecular dynamics (MD) simulations of the ’close to native’ binding mode to investigate the conformational changes associated with receptor activation and uncover the dynamics of GnRH binding.

**Table 1:** Comparison of conserved residues and motifs between Class A GPCRs and GnRH1R. Amino acids are given in the Ballesteros-Weinstein numbering scheme^27^ X.YY where X is the TM number and YY is the residue number according to the most conserved residue.

**Conserved GPCR motif/residue**	**Function in class A GPCRs**	**GnRH1R motif/residue**	**Function in GnRH1R**
D$\phantom{0}^{3.49}$-R$\phantom{0}^{3.50}$-Y$\phantom{0}^{3.51}$ (DRY)	Ionic lock and G-protein interaction site^28,29^; structural and activation of cellular signalling^30,31^	D$\phantom{0}^{3.49}$-R$\phantom{0}^{3.50}$-S$\phantom{0}^{3.51}$ (DRS)	Structural and activation of cellular signalling^30,31^
C$\phantom{0}^{6.47}$-W$\phantom{0}^{6.48}$-x-P$\phantom{0}^{6.50}$-Y$\phantom{0}^{6.51}$ (CWxPY)	Conformation-independent conserved interhelical network^32,33^; conserved water-mediated polar network^34^; forms an exaggerated kink opening G-protein binding pocket^35^	C$\phantom{0}^{6.47}$-W$\phantom{0}^{6.48}$-x-P$\phantom{0}^{6.50}$-Y$\phantom{0}^{6.51}$ (CWxPY)	Structural and ligand binding affinity^32,33^
N$\phantom{0}^{7.49}$-P$\phantom{0}^{7.50}$-x-Y$\phantom{0}^{7.53}$	Conformation-independent conserved interhelical network^32,33^; conserved water-mediated polar network^34^; forms conformation specific interhelical interactions^36^	D$\phantom{0}^{7.49}$-P$\phantom{0}^{7.50}$-L$\phantom{0}^{7.51}$-I$\phantom{0}^{7.52}$-Y$\phantom{0}^{7.53}$ (DPxxY)	Structural, possible Na$\phantom{0}^{+}$ counter ion, activation of cellular signalling^12–15^
P$\phantom{0}^{5.50}$-I$\phantom{0}^{3.40}$-F$\phantom{0}^{6.44}$ (PIF)	Facilitates movement of cytoplasmic TM6 region; part of transmission switch^34,37–39^	P$\phantom{0}^{5.50}$-A$\phantom{0}^{3.40}$-F$\phantom{0}^{6.44}$ (PAF)	Unknown function^11^
Y$\phantom{0}^{5.58}$ (96% of Class A)	Important for receptor activation^34,37–39^	N$\phantom{0}^{5.58}$	Polar interaction with S$\phantom{0}^{3.47}$; tight packing of TM5 with TM3 and TM6^11^
Helix 8 (H8)	Present	Absent	Y$\phantom{0}^{7.53}$ contacts F$\phantom{0}^{1.53}$ and W$\phantom{0}^{1.60}$ in TM1 (due to absence of H8)^11^
X$\phantom{0}^{6.40}$	Short hydrophobic residues	F$\phantom{0}^{6.40}$ (conserved in mammalian GnRH1R)	Important for activation; function unknown^11^
D$\phantom{0}^{2.50}$	Allosteric Na$\phantom{0}^{+}$ binding or activation^32–34,40^	N$\phantom{0}^{2.50}$	Direct polar interaction with D$\phantom{0}^{7.49}$^11^; potential involvement in receptor activation^11^

In the next section we present the results for the activation of GnRH1R following the binding of GnRH found in our MD simulations. We then discuss our results in the context of the current literature, and demonstrate how they explain various observations and help resolve controversies.

## Results

### GnRH1R activation

To explore the potential binding modes of GnRH to the GnRH1R, we performed unbiased computational docking simulations using Rosetta. A total of 27,000 docking simulations were carried out, generating a diverse pool of possible GnRH-GnRH1R binding modes. This pool of candidates was then subjected to a multi-step elimination process, which involved clustering and ranking based on various structural and energy criteria (see Methods and Supplementary Information for details). From this analysis, the two top-scoring binding modes, denoted as ROS-1 and ROS-2 (named after Rosetta Commons), were identified as the most promising candidates.To further evaluate the stability and dynamics of the selected binding modes, we conducted 1.1$\mu$s MD simulations for the ROS-1 and ROS-2 systems. Notably, only the ROS-1 binding mode exhibited receptor activation after 1.0 $\mu$s of simulation, suggesting a possible pathway for GnRH-induced receptor activation. In contrast, the ROS-2 binding mode failed to induce receptor activation, as evidenced by the maintenance of inactive-like TM3-TM6 distances of ≈ 8 Å throughout the simulation (Fig. S1b), compared to the ≈ 4 Å increase observed in ROS-1. The fundamental difference between these binding modes lies in their spatial orientation within the receptor binding pocket: while ROS-1 positions GnRH towards the TM6-TM5-TM4 interface with the peptide’s N-terminus (E1-G6) oriented towards the extracellular region (Fig. S2a, b), ROS-2 adopts a distinctly different configuration with GnRH oriented towards the TM7-TM1-TM2 interface and a more buried positioning of the N-terminal residues (Fig. S2c, d). This altered binding geometry in ROS-2 appears to preclude the formation of the critical intermolecular interactions necessary for inducing the conformational changes that characterise receptor activation, particularly the outward movement of TM6 that is essential for G-protein coupling. Based on these findings, the ROS-1 binding mode was selected for further analysis and characterisation.

**Fig. 1:**
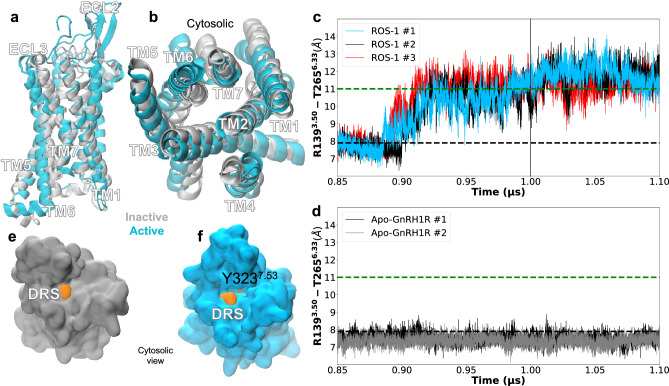
GnRH1R activation dynamics. Side (**a**) and (**b**) cytosolic views of GnRH1R, illustrating the conformational change of TM6. Traces of TM3-TM6 distances over time for (**c**) the active ROS-1 and (**d**) the inactive Apo-GnRH1R system. (**e**) and (**f**) represent the cytosolic site of the receptor in its (**e**) inactive Apo-GnRH1R and (**f**) active ROS-1 conformations. The DRS and Y323$\phantom{0}^{7.53}$ are depicted in orange.

The prevailing activation mechanism observed across Class A GPCRs involves an increase in the TM3-TM6 distance and the disruption of the ionic lock typically found between residues at positions 3.50 and 6.30^41,42^. However, GnRH1R has a polar interaction between R139$\phantom{0}^{3.50}$ an T265$\phantom{0}^{6.33}$ instead, although the active state is expected to show a similar increase in the TM3-TM6 distance^17^. Across the three systems studied herein which involved GnRH, including the two binding modes (ROS-1, ROS-2) and an undocked GnRH-GnRH1R system, only ROS-1 induced receptor activation. Monitoring the TM3-TM6 distance revealed GnRH1R activation at ≈ 1.0 µs in the ROS-1 system. Structural alignment of the active and inactive conformations is presented in Fig. 1. The inactive TM3-TM6 distance from the crystal structure, along with the active-like AlphaFold model, were obtained from the GPCRdb and served as baselines for comparing the inactive and active-like TM distances of the simulated system (ROS-1) (Fig. 1c-d green and black dash lines). Distance analysis showed a ≈ 4 Å increase in the distance between TM3 and TM6 upon activation (Fig. S3), consistent with TM3-TM6 distances observed in other GPCRs.

The conserved residue P282$\phantom{0}^{6.50}$ of the CWxPY motif serves as a pivotal point for the outward bending of TM6 upon activation, a phenomenon observed in numerous GPCRs^43^. It is believed to function as a hinge for the bending of TM6 due to the absence of H-bonds between its backbone nitrogen atom and the carbonyl group of the residue located one helical turn above^43^. This function of P282$\phantom{0}^{6.50}$ in the GnRH1R is observed herein as well, where the outward tilt of TM6 initiates after this residue (Fig. S4). Throughout the simulation period, the intracellular loops (ICLs) of GnRH1R maintain stability, except for ICL3. Notably, ICL3 exhibits an outward turn and becomes a parallel extension of TM5 and TM6 in the active GnRH1R. The substantial rearrangement of ICL3 aligns with previous studies that observe outward movements of this loop^44^.

### The GnRH binding mode

**Fig.2:**
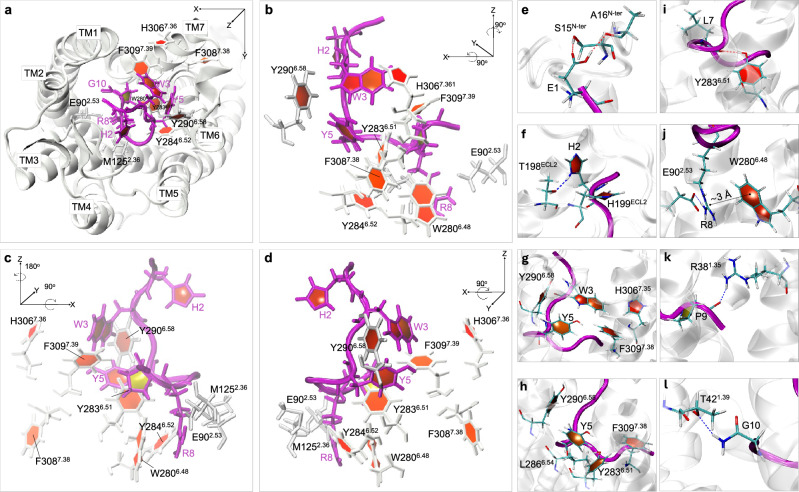
Illustration of the ROS-1 activating binding mode. (**a**) Overview of the binding pose seen from the extracellular space and (**b**) isolated view. (**c**-**j**) Highlighted residues involved in the binding mode, featuring GnRH1R (white) and GnRH (magenta). Snapshots captured at 1.1 $\mu$s.

The active binding mode demonstrates prominent interactions facilitated by a network of aromatic residues, establishing crucial interactions between the N-terminus of GnRH and the aromatic residues of TM6-TM7 in the GnRH1R (Fig. 2).

Specifically, T-shaped $\pi -\pi$ stacking interactions were observed between W3 of GnRH and Y290$\phantom{0}^{6.58}$ and H306$\phantom{0}^{7.35}$ of GnRH1R, complemented by an irregular (I)-shaped $\pi -\pi$ stacking interaction with F309$\phantom{0}^{7.38}$ (Fig. 2b, e, Fig. S5). Furthermore, W3 of GnRH engages in intramolecular T-shaped $\pi -\pi$ stacking interactions with Y5 (Fig. 2b, Fig. S5). Subsequently, Y5 forms $\pi -\pi$ stacking interactions with Y290$\phantom{0}^{6.58}$, Y283$\phantom{0}^{6.51}$, and F309$\phantom{0}^{6.58}$, accompanied by vdW and hydrogen bonding interactions with L286$\phantom{0}^{6.54}$ (Fig. 2b, f). Furthermore, the critical GnRH residue, R8, establishes favourable cation-$\pi$ interactions with the conserved W280$\phantom{0}^{6.48}$ residue of the CWxPY motif (Figure 2h, Fig. S5c), deviating from the previously proposed salt bridge with D302$\phantom{0}^{7.31}$^42,45^which did not form in any of the simulated systems (Fig. S6). In addition to this interaction, low occupancy H-bonds and a low duration salt bridge forms between R8 and E90$\phantom{0}^{2.53}$ (Fig. 2h, Figs. S7, S8a). R8 was also found to form vdW and H-bond interactions with M125$\phantom{0}^{3.36}$ (Fig. 2a, Fig. S7) which has been shown important for GnRH1R activation^11^. Our results parallel recent groundbreaking work^46^ identifying a cryptic pocket in CB1 that accommodates a positively charged guanidinium group. This interaction forces the crucial toggle switch residue W356^6.48^ to adopt an “active-open” conformation through cation-$\pi$ interactions. Similarly, in our system, R8 of GnRH engages with W280^6.48^ through comparable cation-$\pi$ interactions.

Lastly, the N-terminal E1 residue of the modelled GnRH forms H-bonds with the several N-terminal receptor residues including N10^N-ter^ (which reduced GnRH affinity after mutation^47^, S15$\phantom{0}^{N-ter}$, and A16$\phantom{0}^{N-ter}$ (Fig. 2c, Fig. S7). This suggests the potential role of the GnRH1R N-terminus in GnRH binding, challenging previous studies that suggest that the N-terminus co-occupies the orthosteric binding pocket and interacts with agonists and antagonists, but does not participate in GnRH binding^11^. It is important to note, however, that the N-terminus interactions observed in this research are mediated through E1 rather than the native pyroglutamate ring, raising questions about their biological relevance. We also observed that H2 forms T-shaped $\pi -\pi$ stacking and hydrogen bond interactions with H199$\phantom{0}^{ECL2}$ and T198$\phantom{0}^{ECL2}$ (Fig. 2d, Fig. S7). Finally, the C-terminal P9 forms H-bonds with R38$\phantom{0}^{1.35}$ and engages in vdW interactions with Y283$\phantom{0}^{6.51}$ of the CWxPY motif (Fig. 2i, Fig. S7) and G10 forms H-bonds with T42$\phantom{0}^{1.39}$ (Fig. 2j, Fig. S7). Notably, Y283$\phantom{0}^{6.51}$ also forms direct H-bonds with the backbone oxygen of L7 (Fig. 2g, Fig. S7 and Y284$\phantom{0}^{6.52}$) with G6 (Fig. S7).

### CWxPY – DPxxY communication and the water-mediated network

**Fig. 3:**
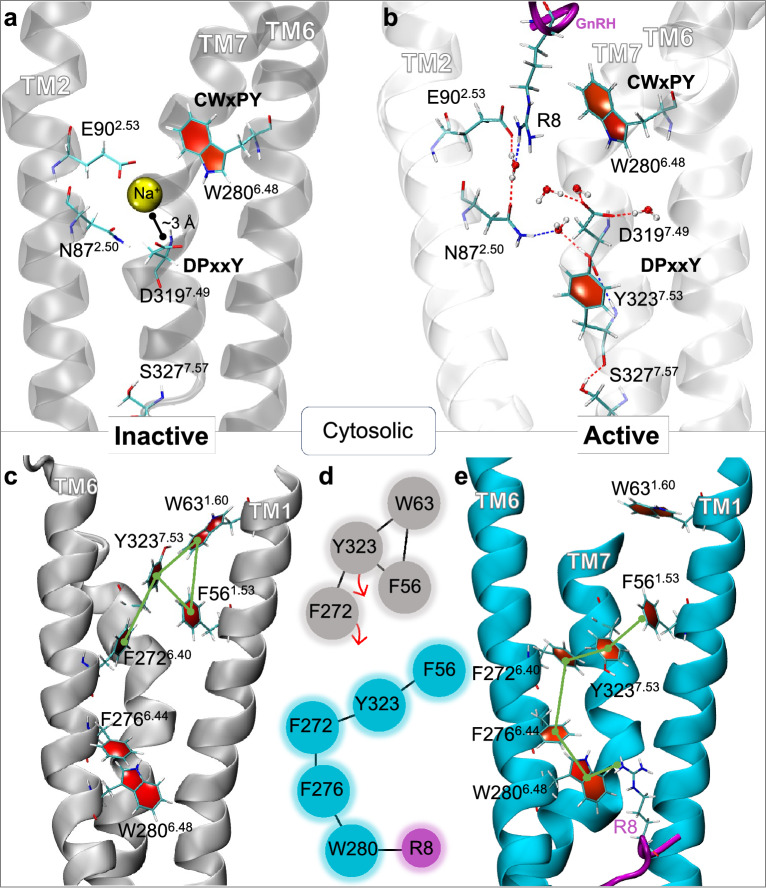
(**a**) Sodium (Na$\phantom{0}^{+}$) binding pocket at the DPxxY motif in the inactive state. (**b**) Water-mediated network interactions between GnRH/CWxPY and E90$\phantom{0}^{2.53}$/N87$\phantom{0}^{2.50}$, as well as the DPxxY motif in the active state. (**c**) and (**e**) Communication pathways between TM1, TM6, and TM7 in both inactive (grey) and active (blue) conformations. In the inactive state, Y323$\phantom{0}^{7.53}$ of the DPxxY motif interacts with TM1 residues F56$\phantom{0}^{1.53}$ and W63$\phantom{0}^{1.60}$. In the active state, the pathway to CWxPY is open, allowing Y323$\phantom{0}^{7.53}$ to communicate with W280$\phantom{0}^{6.48}$/R8 through F272$\phantom{0}^{6.40}$ and F276$\phantom{0}^{6.40}$ of the P$\phantom{0}^{5.50}$A$\phantom{0}^{3.40}$F$\phantom{0}^{6.40}$ motif. (**d**) Contact map illustrating $\pi$-$\pi$ interactions.

GnRH1R presents a negatively charged DPxxY motif^12,13,15^, deviating from the conserved polar NPxxY motif found in other Class A GPCRs^32–34,36^. An intriguing and novel pattern emerges, revealing the communication initiated by R8 of GnRH with the CWxPY and DPxxY motifs through water-mediated H-bonds (Fig. 3b).

Distance analysis of the activation window (1.0-1.1µs) (Figs. S9, S10) demonstrates that R8 forms H-bonds with E90$\phantom{0}^{2.53}$ either directly (38.9–69.8% of simulation time) or through water molecules (29.1–56.5%) across multiple ROS-1 replicas (Fig. S11). This bimodal behaviour indicates dynamic exchange between binding modes, with E90$\phantom{0}^{2.53}$ providing primary anchoring whilst N87$\phantom{0}^{2.50}$ participates in a more flexible relay network. The R8–N87 interaction is predominantly water-mediated (55.7–78.1%), with significant periods of no direct contact (21.9–44.3%) supporting its role in the water-mediated network observed in other GPCRs^11,32,33,40^.

Occupancy analysis (Fig. S12) quantifies network stability. The N87$\phantom{0}^{2.50}$–E90$\phantom{0}^{2.53}$ water bridge maintains exceptionally high occupancy in ROS-1 (83.0–92.0%), reduced by 35% in the inactive Apo-GnRH1R (53.0–57.5%). Water bridges involving R8 with E90 (56.5–82.0%) and N87 (27.0–64.0%) show substantial occupancies in ROS-1, with R8–E90 consistently higher than R8–N87, aligning with more frequent direct contacts. The N87–DPxxY connection show GnRH-dependent enhancement: N87–D319 occupancy increases 2-fold from APO (18.5–35.2%) to ROS-1 (45.6–66.0%), whilst N87–Y323 increases from 23.1–31.8% to 38.9–49.2%, demonstrating how GnRH binding propagates structural changes through organised water networks to the intracellular G protein coupling surface.

Radial distribution function analysis (RDF) (Figs. S13, S14) reveals well-defined hydration shells around E90$\phantom{0}^{2.53}$ and N87$\phantom{0}^{2.50}$ in ROS-1 systems, with sharp first peaks at approximately 2.8 Å. E90$\phantom{0}^{2.53}$ exhibits particularly strong structured hydration with g(r) $\approx$ 23–30, whilst N87$\phantom{0}^{2.50}$ shows g(r) $\approx$ 11–14. In Apo-GnRH1R simulations, both residues show substantially reduced peak heights (E90: g(r) $\approx$ 16–20, N87: g(r) $\approx$ 7–9), indicating less structured hydration in the absence of GnRH. Second hydration shells at approximately 4–5 Å are more pronounced in ROS-1 systems, demonstrating that GnRH binding organises water structure extending beyond the first coordination shell.

Notably, whilst the active GnRH1R conformation forms the R8-CWxPY-DPxxY water-mediated network, D319$\phantom{0}^{7.49}$ of the DPxxY motif captured a sodium atom in one inactive conformation replica (Fig. 3a). The sodium atom inserted into the interhelical space early in the simulation and remained at ≈ 2.5 Å distance from D319$\phantom{0}^{7.49}$ and ≈ 7 Å from W280$\phantom{0}^{6.48}$ throughout the simulation time(Fig. S7b). This confirms the DPxxY motif as a sodium binding site and suggests sodium binding characterises the inactive conformation, with its positive charge replaced by R8 in the active state. Importantly, the sodium ion cannot mediate interactions with N87$\phantom{0}^{2.50}$, whilst R8 interacts with N87$\phantom{0}^{2.50}$ and E90$\phantom{0}^{2.53}$ through water molecules and forms van der Waals interactions with M125$\phantom{0}^{3.36}$. Consequently, M125$\phantom{0}^{3.36}$ interacts with A129$\phantom{0}^{3.40}$ of the PAF motif, located one helical turn above the G-protein binding pocket (DRS motif) (Fig. 3).

The absence of a C-terminal helix in GnRH1R (Table 1) results in tight packing of TM1 and TM7, evident from interactions between F56$\phantom{0}^{1.53}$, W63$\phantom{0}^{1.60}$ and Y323$\phantom{0}^{7.53}$ (DPxxY motif) in the inactive conformation^11^. As TM7 experiences slight inward movement in the active conformation, these $\pi$-$\pi$ interactions are disrupted (Fig. 3c-d, Fig. S5). Y323$\phantom{0}^{7.53}$ rotates towards the centre of the intrahelical bundle, aligning with its observed behaviour in other active GPCRs^36,41^, creating an open $\pi$-$\pi$ network (F56$\phantom{0}^{1.53}$-Y323$\phantom{0}^{7.53}$-F272$\phantom{0}^{2.40}$-F276$\phantom{0}^{2.44}$-W280$\phantom{0}^{2.48}$-R8) that was otherwise closed in the inactive conformation.

### PAF motif and membrane communication

The PIF motif in GPCRs, while not highly conserved, has been proposed to facilitate the outward movement of TM6^34,37–39^. GnRH1R features the P223$\phantom{0}^{5.50}$- A129$\phantom{0}^{3.40}$-F276$\phantom{0}^{6.44}$ (PAF) motif. Comparison of the PAF motif between active and inactive GnRH1R conformations revealed no significant differences except the slight rotation of F276$\phantom{0}^{6.44}$ towards the membrane environment in the active conformation (Fig. S15a). In the active conformation, F276$\phantom{0}^{6.44}$ was observed within 5 Å of a lipid tail after receptor activation, which in turn interacts consistently with R240$\phantom{0}^{5.67}$ at the cytosolic end of TM5 through H-bonds (Fig. S15b,c). This suggests the potential role of F276$\phantom{0}^{6.44}$ in mediating signal through the membrane and stabilising the cytosolic movement of ICL3 and TM6. In 96$\%$ of class A GPCRs, the residue in position 5.58 is a tyrosine and it has been shown to be important for receptor activation^34,37–39^. However, the GnRH1R consists of a polar asparagine in position 5.58. In the inactive GnRH1R, N231$\phantom{0}^{5.58}$ was found at a 6 Å distance from S136$\phantom{0}^{3.47}$ and additionally in close proximity with the same lipid that forms vdW interactions with F276$\phantom{0}^{6.44}$ of the PAF motif and H-bonds with R240$\phantom{0}^{5.67}$ of ICL3 (Fig. S15b-d). This suggests that the unique N231$\phantom{0}^{5.58}$ residue of GnRH1R may be involved in receptor activation through membrane communication.

**Fig. 4:**
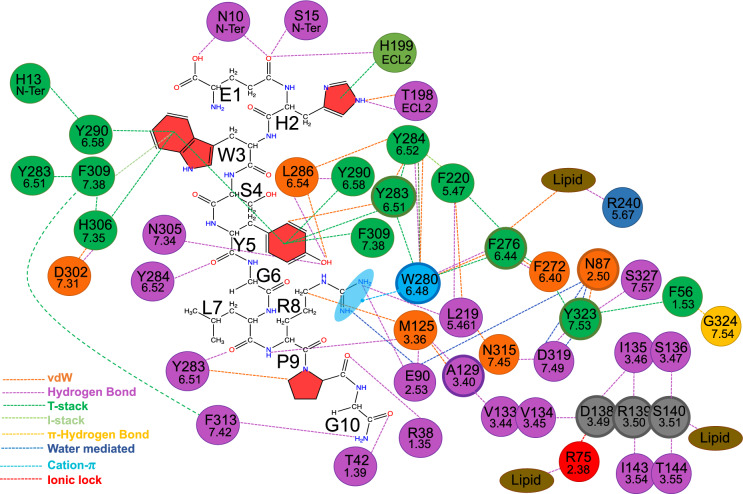
Total GnRH-GnRH1R interactions and communication networks within GnRH1R.

To further investigate the role of lipids in GnRH1R activation, H-bonds between lipids and GnRH1R residues were monitored for the duration of activation (Fig. S16). Notably, the head polar groups of POPC lipids formed high-occupancy H-bonds with polar residues located at the ECLs and ICLs of the receptor where the majority of H-bonds formed through serine, arginine, and lysine residues. On the extracellular side of the receptor, membrane interaction was observed *via* ECL3 residues, and particularly through S301$\phantom{0}^{7.30}$ and R299$\phantom{0}^{ECL3}$. Additionally, residues on the extracellular side of TM5 and ECL2, such as Q204$\phantom{0}^{5.32}$, W206$\phantom{0}^{5.34}$, H207$\phantom{0}^{5.35}$, and S203$\phantom{0}^{ECL2}$, also exhibited high occupancy H-bonds (Fig. S16). Residues at the cytosolic side of TM7, especially Y325$\phantom{0}^{7.55}$ and K233$\phantom{0}^{5.60}$ also participated in H-bonds with lipids. Additionally, ICL3 participated in receptor-lipid H-bonds *via* R240$\phantom{0}^{5.67}$, while ICL2 interacted through R145$\phantom{0}^{3.56}$ (Fig. S16). Furthermore, R75$\phantom{0}^{2.38}$ at the cytosolic end of TM2 forms high occupancy H-bonds with lipids while retaining an ionic lock with D138$\phantom{0}^{3.49}$ of the DRS motif (Figs. S8a, S16). The participation of the DRS residue S140$\phantom{0}^{3.51}$ and DRS-interacting residue R75$\phantom{0}^{2.38}$ in lipid interactions highlights their importance in GnRH1R function and potentially internalisation process (Fig. 4, Fig. S17). Receptor-lipid interactions are also observed at the cytosolic end of TM1, where S55$\phantom{0}^{1.52}$, K59$\phantom{0}^{1.56}$, and K62$\phantom{0}^{1.59}$ form moderate H-bonds with lipids (Fig. S16). Similarly, the cytosolic end of TM2 presents lipid H-bonds with Q61$\phantom{0}^{1.58}$ and K81$\phantom{0}^{2.44}$ (Fig. S16)).

These observations suggest that lipid interactions are not merely structural bystanders but active modulators of GnRH1R dynamics. By stabilising specific transmembrane and intracellular loop conformations, lipids may fine-tune receptor activation thresholds and signalling bias. From a pharmacological perspective, recognising the role of membrane contacts could guide drug design strategies towards allosteric modulators or bitopic ligands that exploit receptor–lipid interfaces, thereby enhancing selectivity and functional control.

### The behaviour of the N-Terminus

**Fig. 5:**
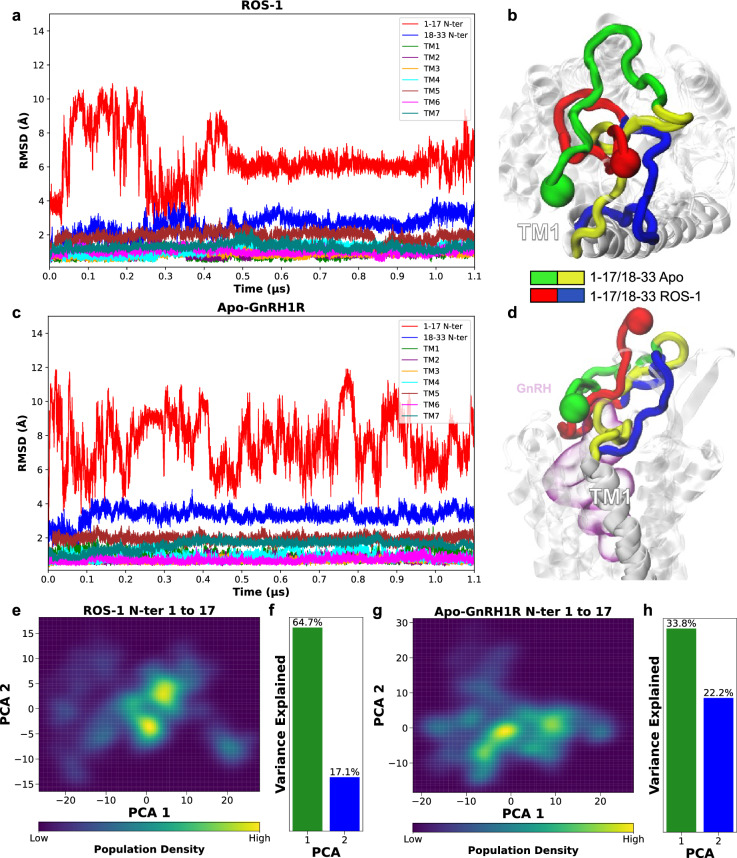
Time evolution of RMSD values for (**a**) ROS-1 and ROS-1 and (**c**) Apo-GnRH1R systems Apo-GnRH1R. The N-terminus is visualised from the extracellular space in. The N-terminus is visualised from the extracellular space in (**b**) and side view in (**d**). In Apo-GnRH, the 1–17 and 18–33 N-terminal residues are depicted in green and yellow, respectively, while in ROS-1, they are represented in red and blue colours. GnRH is displayed in pink in (**d**) for context and has been omitted from (**b**) for clarity. PCA of the N-Terminus residues 1 to 17 and corresponding variance explained for ROS-1 (**e** and **f**) and Apo-GnRH1R systems (**g** and **h**). PCA was conducted for the time frame of 0.5–1.1.5.1 $\mu$s in both systems.

Analysis of N-terminus stability in GnRH1R uncovers its dependence on GnRH binding for conformational equilibration (Fig. 5). RMSD calculations revealed that while the 7TM bundle stabilised within 100–200 ns, the first 17 residues remained highly dynamic, particularly in the Apo-GnRH1R system. In contrast, the ROS-1 complex facilitated the stabilisation of this sequence around 500 ns, suggesting a GnRH-induced conformational locking mechanism (Fig. 5a).

Principal component analysis (PCA) serves as a powerful dimensionality reduction technique that identifies collective motions and dominant conformational modes in biomolecular systems by diagonalising the covariance matrix of atomic coordinates. This approach has proven particularly valuable for characterising protein flexibility and ligand-induced conformational changes in GPCR systems^48^. To further characterise the conformation of the N-terminus we performed PCA for the first 17 residues of the GnRH1R across the active (ROS-1) and inactive (Apo-GnRH1R) simulations. PCA further reinforced these observations, demonstrating greater structural heterogeneity in the Apo-GnRH1R system (Fig. 5g). The ROS-1 system exhibited two distinct clusters, indicative of a more defined conformational space, while the Apo-GnRH1R system showed increased dispersion, reflecting its higher flexibility (Fig. 5e-h).

The population density distributions derived from kernel density estimation of the PC1-PC2 space reveal fundamentally different conformational landscapes between the two systems. In the ROS-1 system, the N-terminal residues 1–17 exhibit a well-defined, concentrated distribution with two prominent high-density regions, suggesting that GnRH binding restricts the conformational sampling to specific, energetically favourable states (Fig. 5e). This clustering behaviour is quantitatively supported by the variance distribution, where PC1 and PC2 capture 64.7 % and 17.1 % of the total variance, respectively, indicating that the essential dynamics can be adequately described by these two dominant modes. The high variance capture by the first two components is characteristic of systems with well-defined conformational preferences, where the majority of structural fluctuations occur along a limited number of collective coordinates.

Conversely, the Apo-GnRH1R system displays a markedly different conformational profile, characterised by a broad, diffuse distribution across the PCA space with multiple overlapping density regions (Fig. 5g). The reduced variance explained by PC1 (33.8 %) and PC2 (22.2 %) indicates that the conformational dynamics cannot be adequately captured by the first two principal components alone, requiring additional higher-order modes to fully characterise the system’s behaviour. This observation is consistent with increased conformational entropy in the app state, where the absence of stabilising interactions between the N-terminus and GnRH allows for extensive sampling of the conformational landscape. The dispersed nature of the population density suggests that the N-terminus adopts multiple conformational substates with comparable populations, rather than converging to distinct, stable conformations.

The contrasting PCA profiles provide mechanistic insights into the role of ligand binding in receptor dynamics. The ligand-induced reduction in conformational dimensionality suggests that GnRH binding not only stabilises the N-terminus but also channels its fluctuations into functionally relevant modes. This conformational restriction may be crucial for receptor activation, as it could facilitate the formation of specific intermolecular contacts required for signal transduction. The ability of PCA to capture these differences demonstrates its utility in quantifying ligand-induced conformational changes and identifying the essential dynamics that govern GPCR function^49^.

Together, these findings suggest that ligand interactions are crucial for stabilising the N-terminal domain, potentially influencing receptor activation and function. PCA analysis reveals that ligand binding fundamentally alters the conformation of the N-terminus, shifting from a broad, multi-state ensemble in the Apo form to a more constrained, bimodal distribution in the ligand-bound state, which may represent functionally distinct conformational states involved in receptor signalling.

## Discussion

### GnRH binding mode

The findings of this study challenge the previously proposed R8-D302^7.31^ salt bridge as a requisite interaction for GnRH1R activation^42^. While earlier computational studies favoured this ionic interaction, our unbiased docking and MD simulations suggest a more intricate binding mechanism. The observed interaction of R8 with deeper intrahelical residues, particularly its cation-$\pi$ interaction with W280^6.48^ of the CWxPY motif (Fig. 2h, Fig. S5c), indicates a potential alternative pathway for receptor activation. This alternative binding mode is further supported by the absence of R8-D302^7.31^ contacts in our docking clusters (Fig. S22), as well as the lack of receptor activation upon the formation of this SB in one replica of the Undocked-GnRH1R simulations (Figs. S1c, S6). These results necessitate a re-evaluation of the prevailing hypothesis, shifting the focus towards alternative interactions that drive GnRH1R activation.

We also provide insights into the structural basis for the binding of different GnRH isoforms. The presence of tyrosine at position 8 in GnRH2, which differs from the arginine found in GnRH1, suggests a distinct yet functionally analogous interaction with the receptor. The ability of GnRH2 to bind both GnRH1R and GnRH2R, despite variations at position 7.31, implies that the SB interaction does not universally dictate GnRH binding. Instead, interactions such as the cation-$\pi$ contact between R8 and W280^6.48^, as well as key $\pi$-$\pi$ stacking interactions involving W3 and Y5 with aromatic residues in TM6 and TM7, emerge as critical determinants of receptor engagement.

Furthermore, the active binding mode identified in this study underscores the relevance of the CWxPY motif and additional residues in stabilising the GnRH1R-GnRH complex. The interactions of Y5 with Y290^6.58^ and F309^7.38^, along with H-bond networks involving E90^2.53^ and M125^3.36^, contribute to a robust binding conformation that facilitates receptor activation. These findings provide a refined perspective on GnRH1R binding and activation, moving beyond the SB hypothesis model to propose a mechanism that integrates a broader network of stabilising interactions.

### GnRH1R activation

GnRH1R activation reveals intricate communication between the CWxPY motif, the DPxxY motif, and GnRH, facilitated by a water-mediated hydrogen bond network (Fig. 3b). Specifically, R8 of GnRH engages in direct or water-mediated H-bonds with E90$\phantom{0}^{2.53}$ and N87$\phantom{0}^{2.50}$, bridging the extracellular and intracellular domains of the receptor. This network extends to the DPxxY motif through additional water-mediated hydrogen bonds with either D319$\phantom{0}^{7.49}$ or Y323$\phantom{0}^{7.53}$. Such interactions highlight the necessity of R8 for effective peptide binding and receptor activation, as it not only stabilises charged interactions but also communicates with transmembrane helices (TM2, TM6, and TM7). Notably, in one replica of the inactive conformation, the DPxxY motif served as a sodium-binding site, with Na$\phantom{0}^+$ maintaining a stable 3 Å distance with D319$\phantom{0}^{7.49}$ (Fig. 3A, Fig. S17b). This suggests that sodium plays a role in stabilising the inactive state, while the positive charge of R8 in GnRH replaces the sodium in the active state, thereby facilitating receptor activation (Fig. 3b,d).

Furthermore, vdW interactions between M125$\phantom{0}^{3.36}$ and A129$\phantom{0}^{3.40}$ in the PAF motif enable communication with the DRS motif (Fig. 4). Hydrogen bond calculations support its role in the water-mediated network of the active state, with N87$\phantom{0}^{2.50}$ forming more hydrogen bonds in the ROS-1 system than in Apo-GnRH1R (Fig. S17a). This network of interactions underscores the significance of the CWxPY and DPxxY motifs in stabilising the active conformation through water-mediated networks, effectively linking GnRH binding to transmembrane helical rearrangements.

The PAF motif (P$\phantom{0}^{5.50}$A$\phantom{0}^{3.40}$F$\phantom{0}^{6.44}$) plays a crucial role in receptor activation, particularly through F276$\phantom{0}^{6.44}$, which undergoes a slight conformational shift towards TM5 and the membrane upon activation (Fig. S16a). This movement places F276$\phantom{0}^{6.44}$ in proximity to a lipid tail, which, in turn, forms stable hydrogen bonds with R240$\phantom{0}^{5.67}$ at the cytosolic end of TM5. Such interactions suggest a possible mechanism by which the receptor communicates with the membrane to facilitate cytosolic domain rearrangements, particularly the outward movement of ICL3 and TM6. Additionally, the unique N231$\phantom{0}^{5.58}$ residue of GnRH1R, which deviates from the more commonly observed tyrosine in Class A GPCRs, participates in lipid interactions (Fig. S15b). In the inactive state, N231$\phantom{0}^{5.58}$ stabilises TM3-TM5 interactions *via* a tight polar contact with S136$\phantom{0}^{3.47}$^11^, whereas in the active state, it moves closer to the lipid tail that also interacts with F276$\phantom{0}^{6.44}$ and R240$\phantom{0}^{5.67}$, potentially aiding in receptor activation *via* membrane-mediated communication (Fig. S15c,d).

To validate our activation analysis beyond the canonical TM3-TM6 distance metric, we performed an evaluation of established GPCR activation signatures across multiple structural domains (Table S1 and Table S2). Our analysis reveals that GnRH1R activation exhibits hallmark features of Class A GPCR activation mechanisms. The observed TM3-TM6 distance increase of +4.34 Å falls within the established range of 3–6 Å reported for other GPCRs^41,50–52^. This primary activation signature is corroborated by significant TM6 rotation (13.7$\phantom{0}^{\circ }$), consistent with the 10–15$\phantom{0}^{\circ }$ rotational changes observed in rhodopsin^52,53^. The conserved molecular switches show expected activation patterns: the DPxxY motif exhibits conformational rearrangement with aspartate displacement (+1.85 Å) and dramatic tyrosine repositioning (+7.95 Å), while the CWxPY motif demonstrates tryptophan toggle switch activation with both translational (+2.72 Å) and rotational (+11.2$\phantom{0}^{\circ }$) components as previously observed^36,41^. Intracellular loop rearrangements show the expected hierarchy, with ICL3 undergoing the largest displacement (+6.39 Å) consistent with its role as the primary G-protein coupling interface^44^, while ICL2 shows more modest reorganisation (+2.07 Å)^54^. The detailed TM6 movement analysis reveals both lateral (+2.45 Å) and vertical (+2.38 Å) displacement^50^.

### GnRH1R-Membrane communication

The interactions between GnRH1R and the surrounding POPC membrane reveal significant lipid-mediated stabilisation of both extracellular and intracellular receptor regions (Fig. S16). Notably, polar residues in the extracellular loops (ECL2 and ECL3) and the cytosolic domains establish high-occupancy hydrogen bonds with POPC head groups. S301$\phantom{0}^{7.30}$ and R299$\phantom{0}^{ECL3}$ exhibit strong lipid interactions, suggesting their role in extracellular domain stabilisation. Similarly, cytosolic residues Y325$\phantom{0}^{7.55}$ and K233$\phantom{0}^{5.60}$, along with R145$\phantom{0}^{3.56}$ in ICL2, participate in lipid interactions, indicating potential involvement in intracellular signalling. The absence of a C-terminal H8 helix in GnRH1R is compensated by lipid interactions involving S140$\phantom{0}^{3.51}$ of the DRS motif and R75$\phantom{0}^{2.38}$ at the cytosolic end of TM2, which retains an ionic lock with D138$\phantom{0}^{3.49}$ (Figs. S8a, S16). These findings suggest that membrane interactions play a crucial role in receptor activation and stabilisation, particularly in the absence of certain structural elements such as Helix 8.

### The behaviour of the N-Terminus

Analysis of N-terminus stability in GnRH1R uncovers its dependence on ligand binding for conformational equilibration (Figure 5). RMSD calculations revealed that while the 7TM bundle stabilised within 100–200 ns, the first 17 residues remained highly dynamic, particularly in the Apo-GnRH1R system. In contrast, the ROS-1 complex facilitated the stabilisation of this sequence around 500 ns, suggesting a ligand-induced conformational locking mechanism (Fig. 5a). The more stable RMSD values observed for the GnRH-bound state highlight the importance of molecular interactions in governing structural dynamics.

PCA further reinforced these observations, demonstrating greater structural heterogeneity in the Apo-GnRH1R system (Fig. 5g). The ROS-1 system exhibited two distinct clusters, indicative of a more defined conformational space, while the Apo-GnRH1R system showed increased dispersion, reflecting its higher flexibility (Fig. 5e-h). The variance captured by PCA1 and PCA2 was notably lower in the GnRH-free state, implying that additional principal components are needed to fully describe its structural landscape. Together, these findings suggest that ligand interactions are crucial for stabilising the N-terminal domain, potentially influencing receptor activation and function.

### Comparative analysis

Lastly, comparative analysis presented in the supplementary Table S3, highlights the significant insights and functional roles of various residues within the GnRH1R, as observed in both existing literature and in this study. For instance, residue R38^1.35^ was previously noted for its interaction with G10 of GnRH, facilitating intrahelical contacts crucial for binding; our simulations corroborate this by demonstrating H-bond formation with the backbone oxygen of P9, indicating a likely interaction with G10 as well. Similarly, N87^2.50^, integral to the water-mediated network, showed a shift in H-bonding dynamics between active and inactive conformations, showcasing its important role in receptor functionality. Notably, residues such as D98^2.60^, and K121^3.32^ displayed conformation-dependent interactions underscoring the importance of structural adaptability in ligand binding and receptor activation. Furthermore, the involvement of residues such as W280^6.48^ and Y283^6.51^ in diverse interaction networks further elucidates the complexity of the receptor activation mechanism, revealing critical insights that advance our understanding of GPCR dynamics and their implications for therapeutic targeting.

The research conducted in this study provides valuable novel insights into the activation mechanism of the GnRH1R. The identified interaction networks, and lipid-mediated communication pathways contribute to our understanding of the structural dynamics and functional implications of GnRH1R activation. Our results help explain previous observations, and consolidate them into a coherent picture of the binding mode and active conformation of the receptor. These findings therefore may enable further research and may inform future drug development efforts targeting this crucial receptor involved in reproductive processes and associated cancers. In particular, the detailed mapping of GnRH1R activation pathways and membrane-mediated modulation offers a structural framework for rational design of GnRH analogues and antagonists, enabling optimisation of ligand-receptor interactions to enhance therapeutic efficacy and selectivity in reproductive hormone-dependent disorders and related cancers.

## Methods

### Computational Docking

The detailed protocols for the preparation, production, and selection of binding modes are described in Supplementary Information of this article (Figs. S4 and S20-23). Briefly, two GnRH-GnRH1R binding modes, namely ROS-1 and ROS-2 (Fig. S2) were selected through a pool of 27,000 decoys generated with the Rosetta Commons FlexPepDocking protocol https://www.rosettacommons.org/docs/latest/application_documentation/docking/flex-pep-dock. The membrane environment was formed using Rosetta MP (Figure S18). To ensure diverse and unbiased docking around the binding pocket^55^, the 15 best scoring poses from the initial 100 poses were used as a template. The template structures underwent further refinement for the production of 1,000 poses per template (total 15,000 decoys). The six best scoring poses in terms of binding energy represented the second template for which additional 2,000 decoys were produced per template (total 12,000 decoys). The resulting binding modes were clustered based on total score and backbone RMSD using the energy-based-clustering protocol implemented with Rosetta https://www.rosettacommons.org/docs/latest/application_documentation/analysis/energy_based_clustering_application ((Fig. S18). Following clustering, energy metrics were calculated using the InterfaceAnalyzer implemented with Rosetta https://www.rosettacommons.org/docs/latest/application_documentation/analysis/interface-analyzer as similarly done in a previous study^56^. As the energy scoring function^57^ employs both physical and statistical metrics, energy metrics are measured in Rosetta Energy Units (REU) which does not translate to actual energy units but provide a good estimation. The developed candidate selection protocol included 5 stages of elimination. The clustering stage resulted in a total of 84 clusters with populations ranging from 2,022 to 50 decoys. In this stage, clusters with population < 50 were eliminated (Stage 1). This yielded 17 clusters for further consideration, from which those with population > 1000 were selected for the next stage, as the native-like binding mode is most likely to exist in a highly populated cluster (Fig. S19, Table S4). Clusters 2, 4, 1 and 5 were selected for further analysis. In the following step, statistical analysis of the Total Score (REU), $\Delta G$ Binding (REU) and $\Delta SASA$ (Å$\phantom{0}^{2}$) of the interface was performed in the form of violin plots. Normality test of the Total Score (REU), $\Delta G$ Binding (REU) and $\Delta SASA$ (Å$\phantom{0}^{2}$) distributions were evaluated using Kolmogorov-Smirnov tests (Fig. S20). Due to mixed populations (Fig. S20, Table S5), non-parametric double-sided Mann-Whitney U tests with Bonferroni correction for multiple comparisons were used using the Scipy.stats library https://docs.scipy.org/doc/scipy/reference/generated/scipy.stats.mannwhitneyu.html with a p-value threshold of 0.05 (p < 0.05 indicates statistically different populations in terms of the metric of interest). Effect size (ES) calculations were also performed to estimate the actual difference of the metrics within the clusters under evaluation as follows:

$$\begin{aligned} EF = 1 - \frac{2 \cdot U}{n_1 - n_2} \end{aligned}$$

where U is the Mann-Whitney statistic and $n_{1}$, $n_{2}$ is the data of populations (clusters) 1 and 2. In this stage binding modes with Total Score > −620 REU (criterion selected based on the Total Score prior to docking) and $\Delta G$ Binding > 0 REU were eliminated (Fig. S21, Table S6).

In Stage 2, contact analysis of the successful clusters was employed. Contacts (namely general contacts) were calculated based of C$\beta$-C$\beta$ carbons (C$\alpha$ for glycine) between GnRH and GnRH1R under a 5 Å cutoff distance. Binding modes that did not form at least one general contact under the specified criteria were eliminated (Fig. S22, Table 2). In Stage 3, binding modes were selected only if GnRH formed contacts with experimentally important GnRH1R residues (as shown in mutation experiments^11^ (namely important contacts) and if $\Delta G$ Binding < −20 REU. Important contacts constituted between GnRH residues^11^ and L23$\phantom{0}^{N-ter}$, T32$\phantom{0}^{1.29}$, R38$\phantom{0}^{1.35}$, D98$\phantom{0}^{2.60}$, N102$\phantom{0}^{2.64}$, K121$\phantom{0}^{3.32}$, Q174$\phantom{0}^{4.61}$, F178$\phantom{0}^{4.65}$, W280$\phantom{0}^{6.48}$, Y283$\phantom{0}^{6.51}$, Y284$\phantom{0}^{6.52}$, L286$\phantom{0}^{6.54}$, Y290$\phantom{0}^{6.58}$, D302$\phantom{0}^{7.31}$, H306$\phantom{0}^{7.35}$, F308$\phantom{0}^{7.37}$ and F309$\phantom{0}^{7.38}$. In the final stage of binding mode selection, the two best scoring poses (ROS-1 and ROS-2) that presented the highest number of important contacts and the lowest $\Delta G$ Binding were selected for further investigation with MD simulations (Fig. S2 and S23). To ensure the selection of biologically relevant binding modes, those that did not satisfy the following criteria in any stage were eliminated:

**Table 2:** Elimination criteria applied during the candidate selection protocol.

Elimination Criteria	Stage 1 (Clustering results)	Stage 2 (Cluster statistical analysis)	Stage 3 (Cluster contact analysis)	Stage 4 (Candidate selection)	Selection
Cluster population	< 50	< 1,000	-	-	-
Total Score (REU)	-	> −620	-	-	-
$\Delta G$ Binding (REU)	-	> 0	-	> −20	Lowest
General contacts	-	-	< 1	-	-
Important contacts	-	-	-	< 1	Highest

Prior research on the GnRH1R-GnRH binding suggested no involvement of the N-terminus in GnRH binding^11^. The decision was made to exclude the initial eleven N-terminal residues from consideration in computational docking simulations due to their strong interference with the docking process. This deliberate choice not only mitigates interference with GnRH binding but also preserves the structural integrity of the disulphide bridge between C14 and C200 in the GnRH1R. In this case, the AlphaFold2-predicted N-terminus was fully disordered, and retained no stable structure prior to equilibration (Fig. S24). Moreover, the receptor backbone was held fixed during docking, allowing only side-chain flexibility, thus ensuring that exclusion of the N-terminus did not impact the conformation of the orthosteric pocket (Fig. S25). After docking, the full-length receptor (including the N-terminus) was reintroduced for all subsequent MD simulations. Notably, the N-terminal region only began to adopt a defined conformation after several hundred nanoseconds of simulation (Fig. 5), at which point it was observed to form transient contacts with the ligand (Figs. 2 and 4). In MD simulations where the N-terminus was included, a strong interference with GnRH impeded the binding dynamics of the peptide. For this reason, the N-terminus was omitted from MD systems where GnRH was left to roam the extracellular space for unconstrained binding (undocked GnRH–GnRH1R systems). However, the N-terminus was included for the MD simulations of Rosetta-docked structures and the Apo-GnRH1R systems.

### MD simulations

The selected ROS-1 and ROS-2 GnRH-GnRH1R binding modes from the docking simulations were subjected to microsecond-timescale MD simulations (the duration of each independent replica was equal to 1.1$\mu$s) to impartially assess their stability and capacity to activate the receptor (Figs. S1a, b, and S2). Additional MD simulations were performed on an unbound GnRH-GnRH1R system to investigate the possibility of GnRH binding and receptor activation naturally (GnRH was placed 20 Å away from GnRH1R) (Fig. S1c). Simulations were also conducted for the GnRH1R in the absence of GnRH (Apo-GnRH1R) to develop an equilibrated model of the inactive receptor state (Fig. S1d). All systems for simulation were constructed underaccepted protocols^58,59^ using the CHARMM-GUI interface https://charmm-gui.org. Briefly, subsequent rounds of equilibration using slow-releasing restraints was conducted in NVT-NPT ensembles at a 1.0 fs timestep for a total of 1,525 ps. Unrestrained production runs were performed in the NPT ensemble at a 2.0 fs timestep using the leap-frog algorithm. The Nosé-Hoover Langevin-piston barostat and Langevin temperature coupling were employed to control the NPT conditions. PME were used to compute long-range electrostatic interaction and periodic boundary conditions were applied. Two fully independent replica simulations were initiated for each system. Furthermore, for the ROS-1 binding mode that induced activation, three dependent replica simulations were performed. The dependent replicas initiated from time equal to 0.85$\mu$s of the original ROS-1 simulation. At this time point, the receptor is still in its inactive conformation with TM3-TM6 distance of $\approx$ 7.5 Å (Fig. S1a). This allows the statistical reliability of the activation process to be assessed using reasonable computational resources.

#### Receptor Activation Analysis

To assess GnRH1R activation, TM3-TM6 distances were calculated over the trajectories based on the distance between R139$\phantom{0}^{3.50}$ and T265$\phantom{0}^{6.33}$. For reference, the inactive crystal structure of the GnRH1R see here has a TM3-TM6 distance of 7.9 Å, while active-like GnRH1R AlphaFold model see here show a distance of 11 Å (Fig. S1). TM3-TM6 distances were calculated using MDTraj https://www.mdtraj.org/1.9.8.dev0/index.html. Further analyses focused exclusively on the activated GnRH1R portion of the trajectories (between 1.0 and 1.1$\mu$s), which yielded 100 ns of activated GnRH1R simulation time. Hydrogen bonds between GnRH-GnRH1R and GnRH1R-lipids were calculated using a 3.5 Å N-O cutoff distance and 20$\phantom{0}^{\circ }$ angle cutoff using the VMD plugin ‘Hydrogen bonds’ https://www.ks.uiuc.edu/Research/vmd/plugins/hbonds/. Complete intermolecular and intramolecular interactions including $\pi -\pi$, vdW, ionic, $\pi$-hydrogen bonds, and hydrogen bond interactions were generated using the RING webserver (https://ring.biocomputingup.it) over the active GnRH1R conformation timeframe (0.1–0.11.1.11$\mu$s, 1 frame/ns yielded 100 frames for analysis) under strict cutoffs (Figs. S5, S26). For GnRH, we utilised the crystal structure deposited under PDBID:1YY1, with pyroglutamic acid modified to protonated glutamic acid as previously described by Connell et al^60^. For the GnRH1R we used an AlphaFold-predicted model based on the inactive crystal structure (PDBID:7BR3) reported by Yan et al^11^. We acknowledge that the AlphaFold model used as our starting structure represents limitations inherent to computational predictions for GPCRs. AlphaFold models are predominantly trained on inactive GPCR conformations and may not accurately capture active state features, particularly in dynamic regions such as the cytoplasmic domains and intracellular loops that undergo major rearrangements during activation^61,62^. The AlphaFold GnRH1R model shows reduced confidence scores (pLDDT < 70) in the intracellular loop regions critical for G protein coupling. However, our extensive molecular dynamics simulations were designed to test whether ligand binding could induce conformational transitions from this inactive-like starting state, and the observed canonical activation signatures validate the robustness of our approach despite these initial structural limitations.

#### Trajectory analysis

Backbone RMSD calculations were performed using the VMD plugin https://www.ks.uiuc.edu/Research/vmd/plugins/rmsdtt/‘RMSD Trajectory Tool’. Salt bridge analysis was conducted using the VMD plugin ‘Salt Bridges’ https://www.ks.uiuc.edu/Research/vmd/plugins/saltbr/using a 3.5 Å N-O distance cutoff and the duration of each salt bridge was calculated and plotted through custom Python scripts.

#### Principal Component Analysis

Principal component analysis was performed to characterise the essential dynamics of the N-terminal residues 1–17 over the time frame of 0.5–1.1 $\mu$s. Atomic coordinates of the selected residues were extracted from the trajectory and reshaped into a matrix where each row represents a frame and each column represents a Cartesian coordinate. PCA was conducted by computing the covariance matrix of the coordinate data followed by eigenvalue decomposition to identify the principal components that capture the largest variance in the conformational ensemble. The analysis was performed using the scikit-learn PCA implementation, with the first two principal components (PC1 and PC2) retained for visualisation and analysis. Population density distributions in the PC1–PC2 space were calculated using Gaussian kernel density estimation (KDE) with the scipy.stats.gaussian_kde function to quantify conformational sampling patterns. The variance explained by each principal component was computed as the ratio of individual eigenvalues to the sum of all eigenvalues, providing a measure of the relative importance of each mode in describing the system’s dynamics.

#### Water-mediated network analysis

Water-mediated hydrogen bond networks were identified and quantified using a systematic distance-based approach implemented in MDTraj. Water bridges were defined as water molecules simultaneously forming hydrogen bonds with two protein residues or between protein residues and ligand atoms. The criteria for hydrogen bond formation included: (i) a maximum distance of 3.5 Å between heavy atoms (donor/acceptor) and water oxygen atoms, and (ii) geometric constraints ensuring proper hydrogen bonding orientation.

For each frame of the MD trajectories, all water molecules within the defined cutoff distance of target residues (N87$\phantom{0}^{2.50}$, E90$\phantom{0}^{2.53}$, D319$\phantom{0}^{7.49}$, Y323$\phantom{0}^{7.53}$, and R8 of GnRH) were identified. Water bridge occupancy was calculated as the fraction of frames in which a specific residue pair was connected *via* at least one bridging water molecule over the entire simulation trajectory. Only water bridges with occupancy $\ge$ 10% were considered significant for analysis. Temporal evolution of water bridge formation was analysed using a sliding window approach with smoothing applied *via* moving averages (window size = 50 frames) to identify persistent network patterns. Distance analysis between R8 and key residues (E90$\phantom{0}^{2.53}$/N87$\phantom{0}^{2.50}$) was performed to distinguish direct versus water-mediated interactions, with direct contact defined as heavy atom distances $\le$ 3.5 Å. The analysis was performed using custom Python scripts.

## Supplementary Information

Supplementary Information.

## Data Availability

The datasets generated and/or analysed during the current study are available in the GnRH1R repository (https://github. com/ePaspali/GnRH1R) and in the Supplementary information.
