# Supplementary material for: Computational modelling reveals novel insights into GnRH receptor activation and binding dynamics
Source: Sci Rep. 2025 Nov 27;15:42479. doi: 10.1038/s41598-025-26518-8 (PMC12660792; doi:10.1038/s41598-025-26518-8)
Supplement: Supplementary file 1 — Supplementary Information. [file 41598_2025_26518_MOESM1_ESM.pdf]

# Supplementary Information: Computational modelling reveals novel insights into GnRH receptor activation and binding dynamics

**Elpiniki Paspali[1], Valerie Anne Ferro[2], Karina Kubiak-Ossowska[3], and Paul Alexander Mulheran[1,*]**

[1]University of Strathclyde, Chemical & Process Engineering, Glasgow, G1 1XL, UK
[2]University of Strathclyde, Strathclyde Institute of Pharmacy & Biomedical Sciences, Glasgow, G4 0RE, UK
[3]University of Strathclyde, Department of Physics/ARCHIE-WeSt, Glasgow G4 0NG, UK
[*]paul.mulheran@strath.ac.uk

## ABSTRACT

Gonadotrophin-releasing hormone (GnRH) regulates the mammalian reproductive system by binding to its receptor (GnRH1R) and is a target for treating reproductive hormone-dependent disorders and cancers. While the inactive structure of GnRH1R is known, the active conformation and GnRH binding mode that lead to receptor activation are not fully understood.The mechanism of GnRH-induced receptor activation remains poorly understood due to the absence of experimental structures of the active GnRH1R-GnRH complex. To address this gap, we employed computational docking simulations using Rosetta, coupled with a custom Python-based elimination protocol, to identify near-native binding poses. This approach yielded two top-ranked candidates, ROS-1 and ROS-2. Molecular dynamics simulations revealed that ROS-1 induced GnRH1R activation within 1.0 $\mu$s, characterised by an $\approx$ 4 Å outward displacement of the cytoplasmic ends of TM3 and TM6. Key interactions included $\pi-\pi$ stacking between GnRH residues and GnRH1R (notably Y5 with Y283$^{6.51}$, Y290$^{6.58}$, and F309$^{7.38}$) and hydrogen bonds with L286$^{6.54}$. Intramolecular $\pi-\pi$ interactions within GnRH (Y5 and W3) also played a significant role. Two main communication pathways initiated from R8 of GnRH were identified. R8 formed cation-$\pi$ interactions with W280$^{6.48}$ and communicated with N87$^{2.50}$ and the DPxxY motif *via* water-mediated hydrogen bonds. Additional interactions involved M125$^{3.36}$ and the PAF and DRS motifs, which are critical for receptor activation. In the active state, certain characteristic $\pi-\pi$ interactions observed in the inactive conformation were absent due to the inward movement of TM7. Finally, the GnRH1R communicated with lipids through hydrogen bonds involving R240$^{5.67}$, R75$^{2.38}$, and S140$^{3.51}$. This study provides insights into the active conformation and binding interactions of the GnRH-GnRH1R complex, advancing our understanding of the process by providing a coherent picture that consolidates previous interpretations, thereby paving the way to better therapeutic applications.

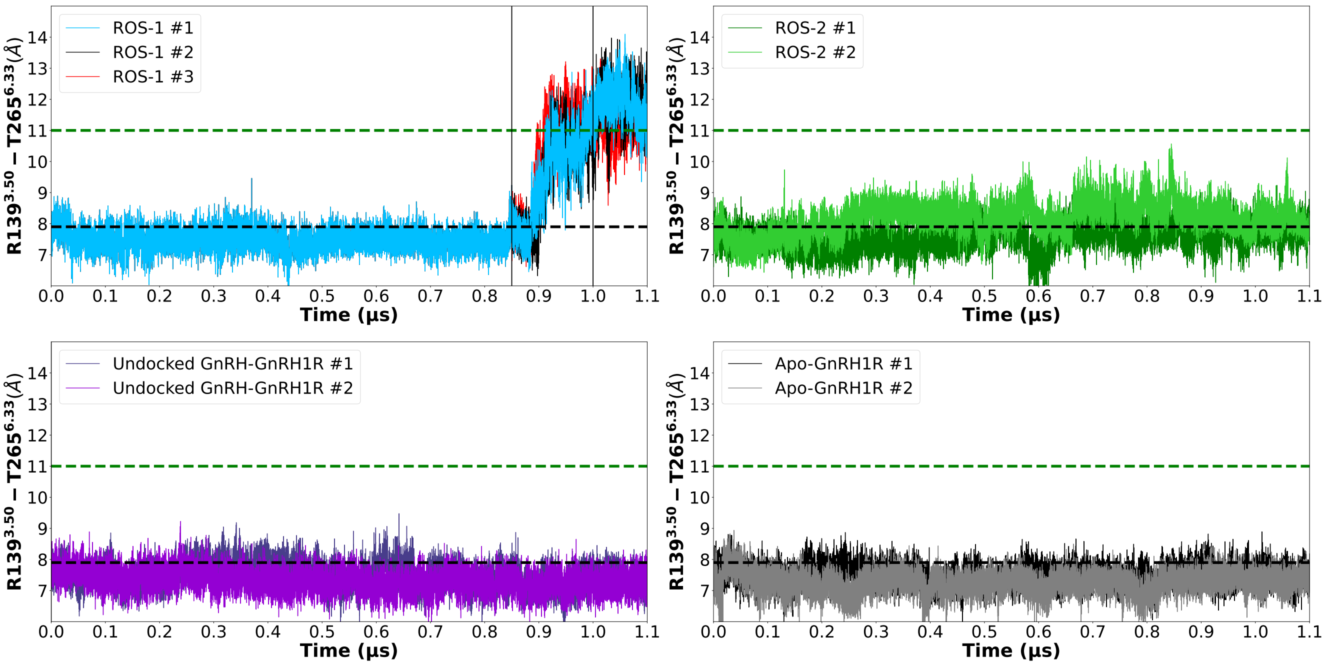


**Figure S1.** Temporal evolution of TM3-TM6 distances measured by R139$^{3.50}$-T265$^{6.33}$ C$\alpha$ Carbons. a) Dynamics of ROS-1 activation, showcasing TM3-TM6 distance stabilisation at $\sim$1.0 µs. Analysis focused on the activated receptor during the 1.0 µs to 1.1 µs timeframe, with replica initiation at 0.85 µs. b) ROS-2 replicas. c) The undocked GnRH-GnRH1R systems. D) The Apo-GnRH1R system. Green line: active-like AlphaFold predicted TM3-TM6 distance; Black line: inactive GnRH1R crystal TM3-TM6 distance.

**Table S1.** GPCR activation metrics for GnRH1R transition from inactive to active state (ROS-1 replica 1 and APO-GnRH1R replica 1)

| Category | Metric | Inactive | Active | Change |
|---|---|---|---|---|
| Transmembrane Distances | TM3–TM6 Distance | 7.90 Å | 12.24 Å | +4.34 Å |
| | TM3–TM7 Distance | 17.88 Å | 18.67 Å | +0.80 Å |
| | TM6–TM7 Distance | 13.10 Å | 15.48 Å | +2.38 Å |
| Transmembrane Rotations | TM3 Rotation | 0° (ref) | 11.6° | +11.6° |
| | TM6 Rotation | 0° (ref) | 13.7° | +13.7° |
| DPxxY Motif Analysis | Center of Mass | 0 Å (ref) | 1.95 Å | +1.95 Å |
| | Asp Carboxyl | 0 Å (ref) | 1.85 Å | +1.85 Å |
| | Tyr Aromatic Ring | 0 Å (ref) | 7.95 Å | +7.95 Å |
| CWxP Motif Analysis | Trp Indole Displacement | 0 Å (ref) | 2.72 Å | +2.72 Å |
| | Trp Ring Rotation | 0° (ref) | 11.2° | +11.2° |
| | Cys Sulfur | 0 Å (ref) | 1.61 Å | +1.61 Å |
| Intracellular Loops | ICL2 Displacement | 0 Å (ref) | 2.07 Å | +2.07 Å |
| | ICL3 Displacement | 0 Å (ref) | 6.39 Å | +6.39 Å |
| TM6 Detailed Movement | Lateral Displacement | 0 Å (ref) | 2.45 Å | +2.45 Å |
| | Vertical Displacement | 0 Å (ref) | 2.38 Å | +2.38 Å |

**Table S2.** Key activation signatures

| Activation Metric | Magnitude | Interpretation |
|---|---|---|
| TM3–TM6 Distance Increase | +4.34 Å | Major structural opening |
| TM6 Rotation | 13.7° | Significant helical reorientation |
| TM6 Lateral Displacement | 2.45 Å | Outward movement (activation) |
| ICL3 Rearrangement | 6.39 Å | G-protein coupling interface |
| Tyr Ring Repositioning | 7.95 Å | Major side chain switch |
| Trp Toggle Switch | 11.2° | Allosteric coupling mechanism |

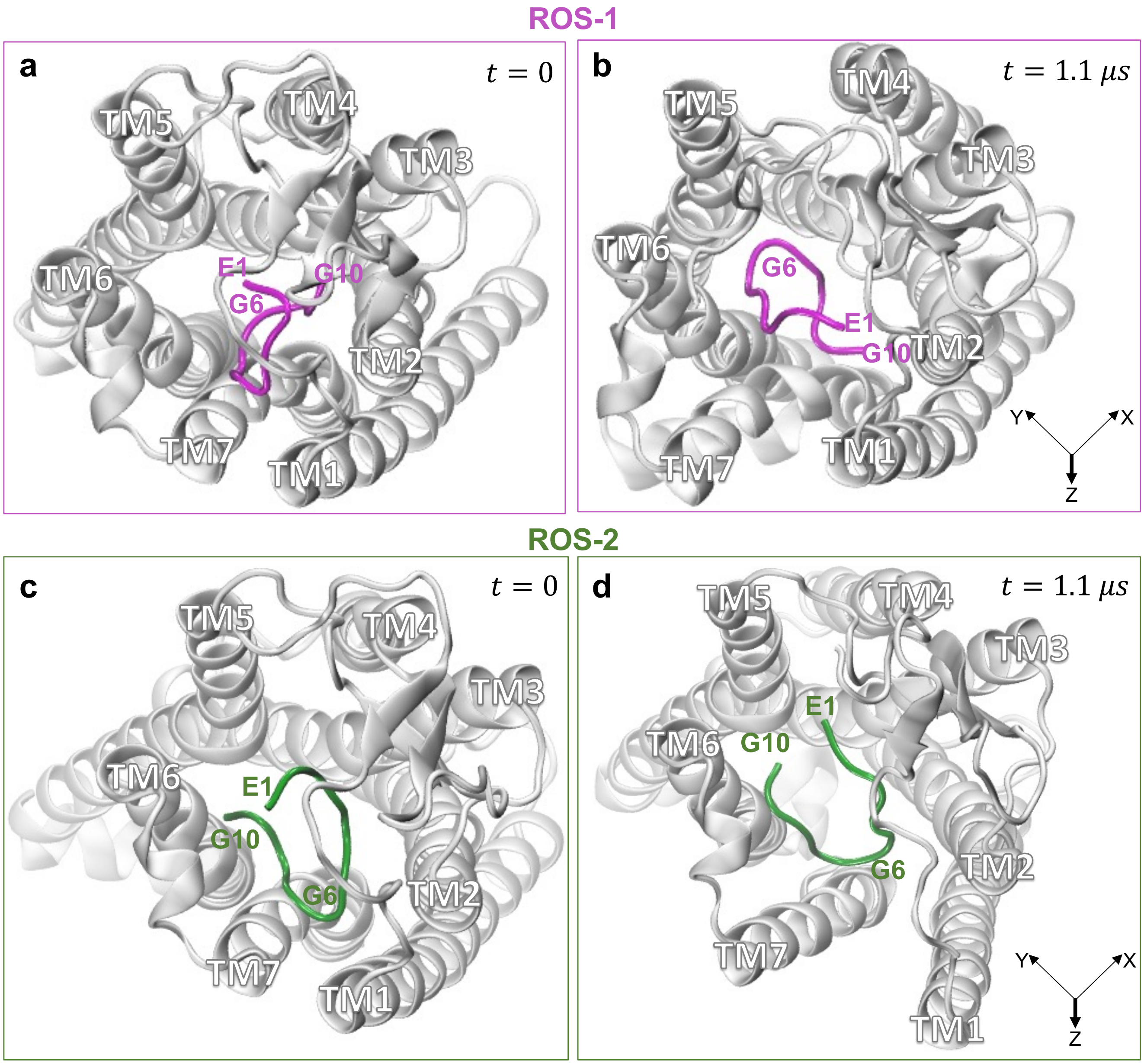


**Figure S2.** Evolution of ROS-1 and ROS-2 binding modes through MD simulations. a) and b) depict the initial and final (1.1 μs) binding modes of ROS-1, respectively. Similarly, c) and d) illustrate the initial and final (1.1 μs) binding modes of ROS-2. The N-terminus, C-terminus, and the central G6 residue of GnRH are denoted.

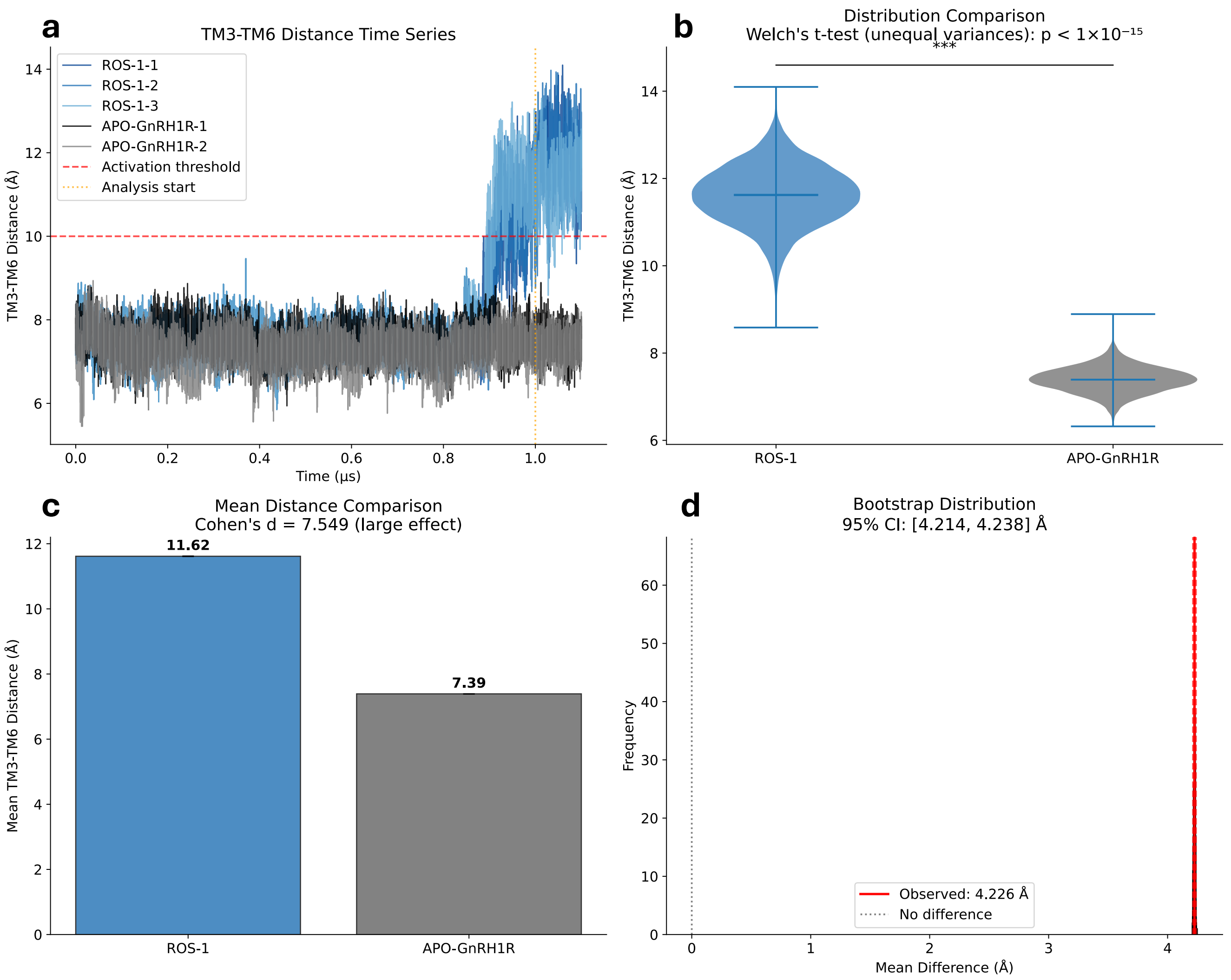


**Figure S3.** Statistical validation of GnRH1R activation through TM3-TM6 distance analysis. a) Time-series analysis of TM3-TM6 distance over 1.1 $\mu$s MD simulations. ROS-1 replicates (blue shades: ROS-1-1, ROS-1-2, ROS-1-3) show activation after $\sim$1.0 $\mu$s, crossing the 10 Å activation threshold (red dashed line), while APO-GnRH1R replicates (black, grey) remain at inactive distances throughout the simulation. Analysis was conducted after equilibration at 1.0 $\mu$s (yellow dotted line). b) Violin plot comparison of TM3-TM6 distance distributions after equilibration, showing complete separation between conditions with no distributional overlap. Statistical significance determined by Welch's t-test accounting for unequal variances ($p < 1\times10^{-15}$). c) Mean distance comparison demonstrating a 4.23 Å difference between conditions (11.62 $\pm$ SEM vs 7.39 $\pm$ SEM Å) with Cohen's d = 7.549, indicating an extremely large effect size. Error bars represent standard error of the mean. d) Bootstrap analysis (n = 1000 iterations) of mean differences showing the observed difference (4.226 Å, red line) with 95% confidence interval [4.214, 4.238] Å. The narrow confidence interval and complete separation from zero (dotted line) confirms the robustness of the activation signature. The $\sim$4.2 Å TM3-TM6 distance increase represents the canonical GPCR activation hallmark, consistent with established activation signatures in other Class A GPCRs.

To validate the TM3-TM6 distance as a reliable activation metric, we employed multiple complementary statistical tests. The time-series analysis (Figure S19a) shows ROS-1 replicates transitioning from inactive ($\sim$8 Å) to active (>12 Å) distances after 0.9 $\mu$s, while APO-GnRH1R replicates remained stable at 7-8 Å throughout the simulation. Statistical analysis of equilibrated data (after 1.0 $\mu$s) used Welch's t-test to accommodate unequal variances between conditions (ROS-1: $\sigma$ = 0.682 Å; APO-GnRH1R: $\sigma$ = 0.293 Å). Both Welch's t-test and Mann-Whitney U test yielded $p < 1\times10^{-15}$ with sample sizes of n = 15,003 and n = 10,002, respectively. Cohen's d = 7.549 indicates an extraordinarily large effect size, with group means separated by more than seven standard deviations. Bootstrap analysis (Figure S19d) generated a narrow 95% confidence interval [4.214, 4.238] Å for the 4.23 Å mean difference, confirming robustness independent of distributional assumptions. The

observed ∼4.2 Å TM3-TM6 distance increase aligns with the canonical GPCR activation signature range of 3-6 Å reported for $\beta_2$-adrenergic receptor, rhodopsin, and $A_2A$ adenosine receptor, confirming that GnRH binding in the ROS-1 configuration induces the structural transitions characteristic of Class A GPCR activation.

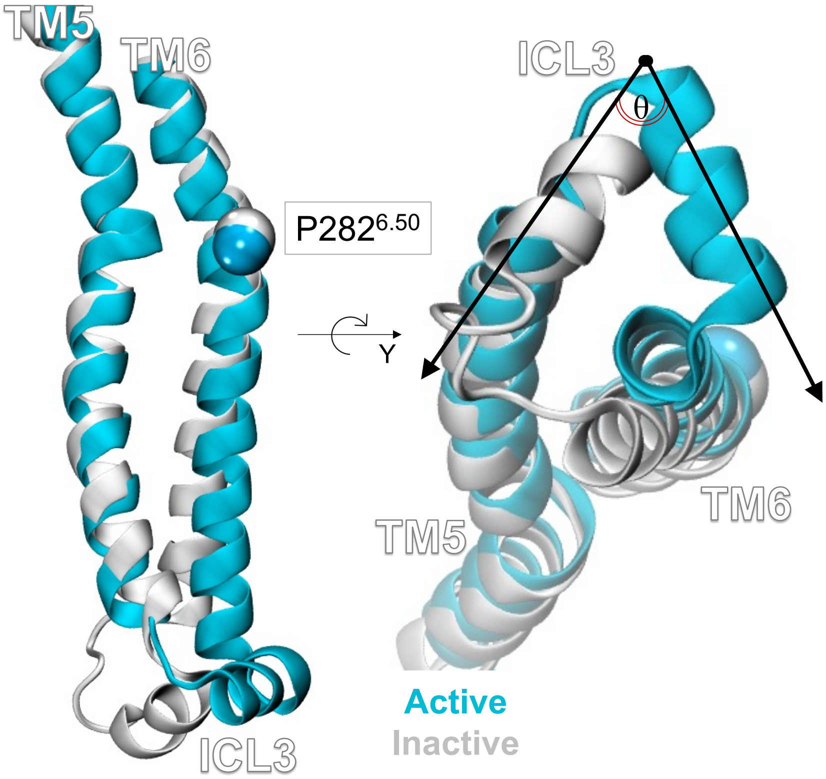


**Figure S4.** Conformation of ICL3 and TM6 kink following P282$^{6.50}$ in the active conformation (blue). ICL3 and TM6 conformation of the inactive conformation is presented in white colour.

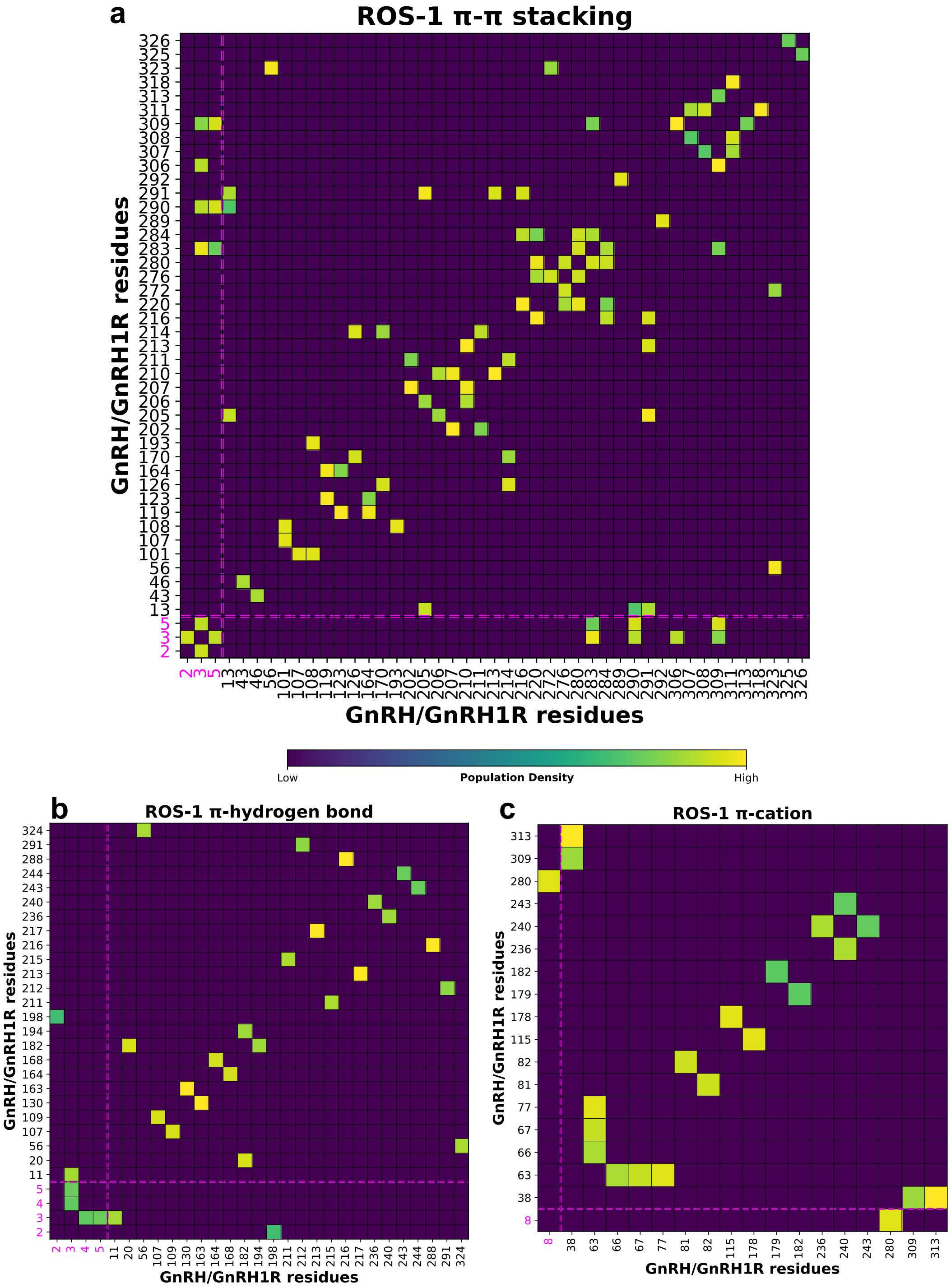


**Figure S5.** a) $\pi - \pi$ stacking interaction analysis in the ROS-1 system throughout the activated simulation. Pink numbers and lines represent GnRH residues and its intramolecular or intermolecular interactions. b) $\pi$-hydrogen and c) $\pi$-cation interactions. Calculations conducted using RING (https://ring.biocomputingup.it).

a

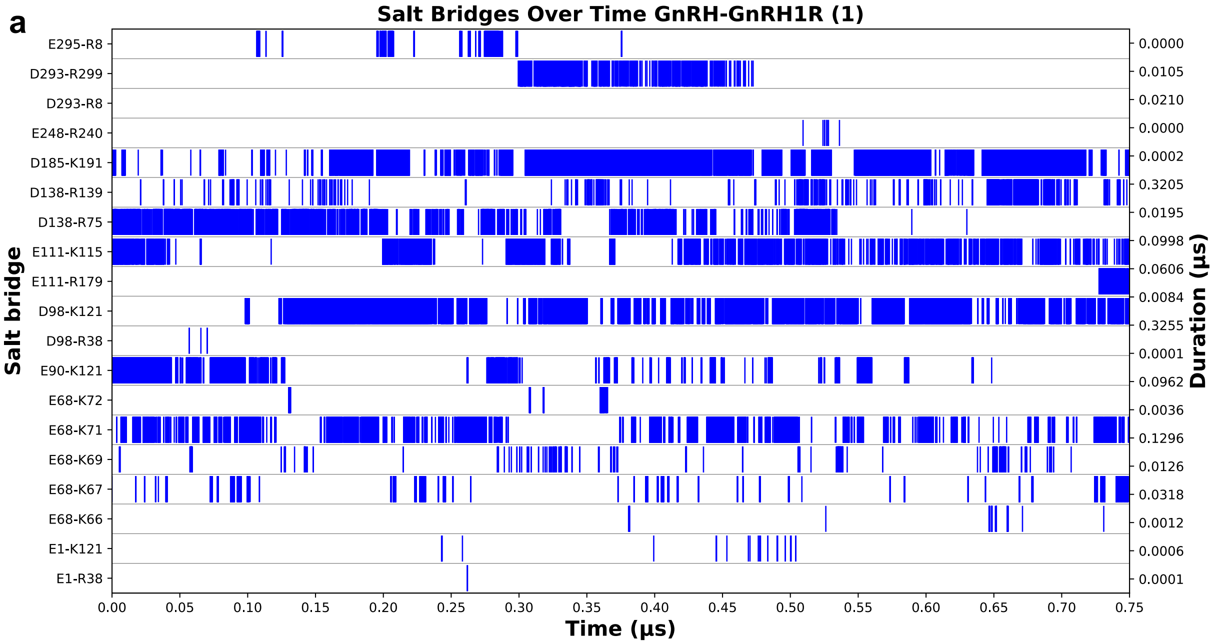


b

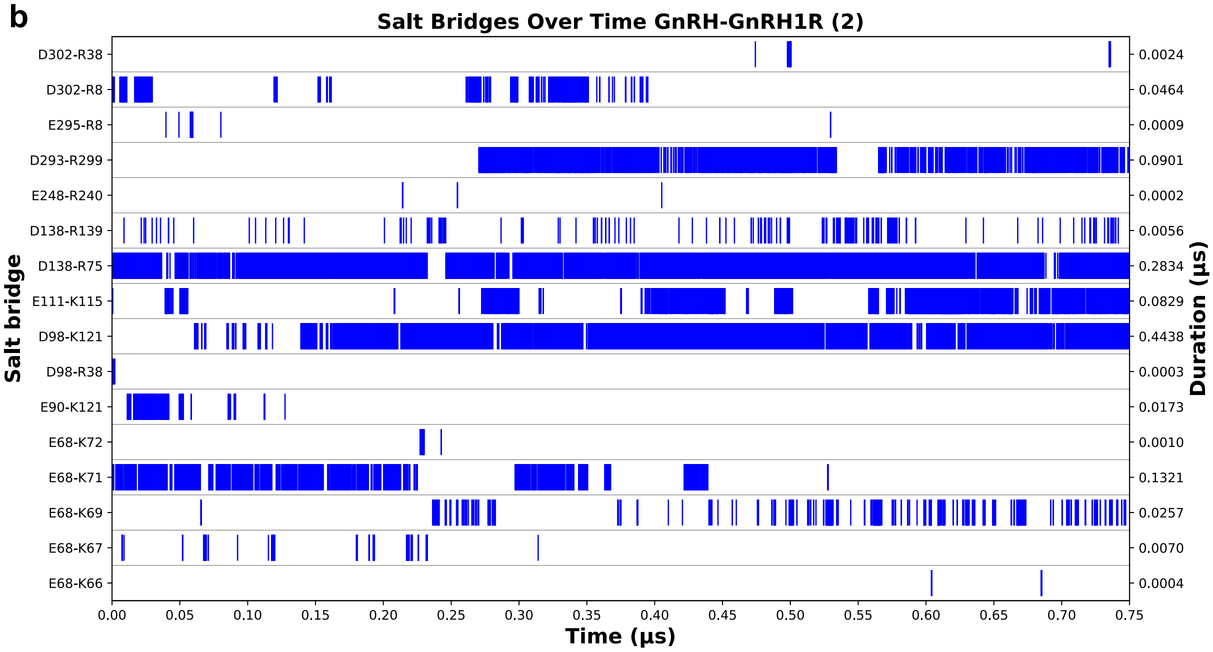


**Figure S6.** Salt bridges over time for the GnRH-GnRH1R systems

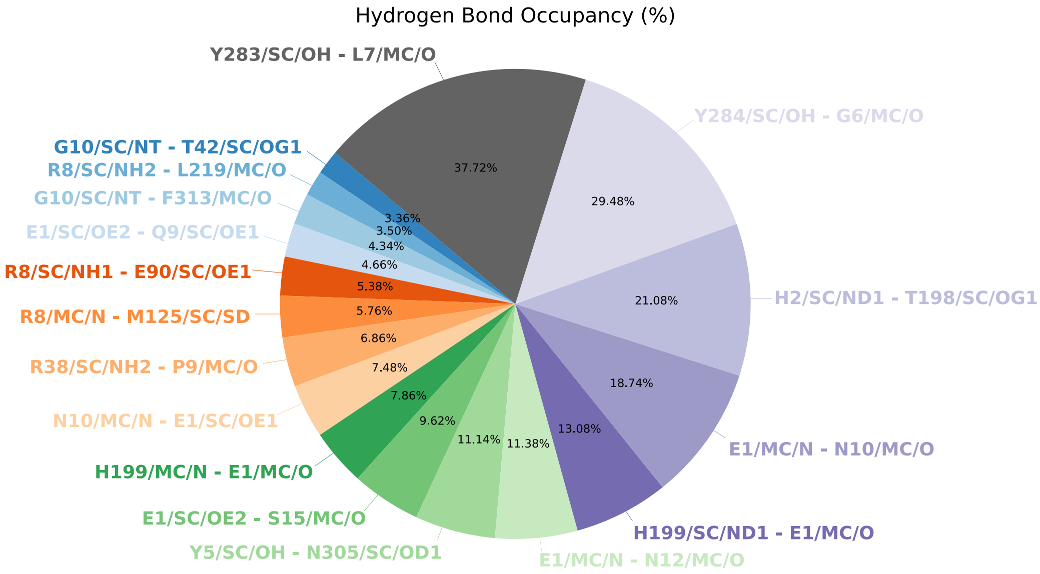


**Figure S7.** H-bond occupancy between GnRH and GnRH1R in the final 100 ns of activation (1.0 µs to 1.1 µs). H-bonds with occupancy $< 3\%$ are omitted for clarity. Residue order follows the Donor-Acceptor format, with MC indicating main chain and SC indicating side chain. Atoms participating in the H-bonds are listed at the end of each label.

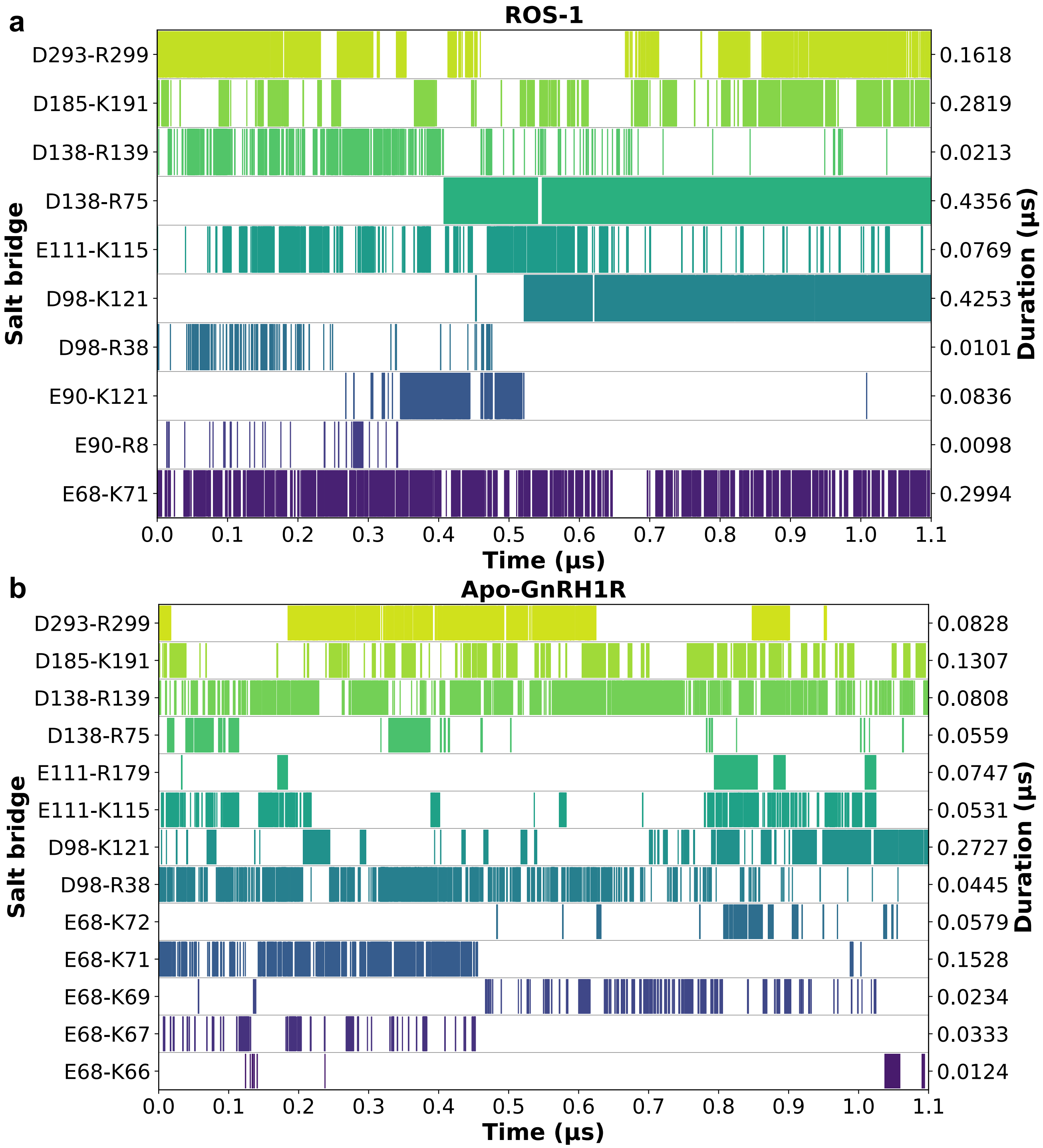


**Figure S8.** Analysis of salt bridge formation and evolution in a) ROS-1 and b) Apo-GnRH1R systems throughout the simulation.

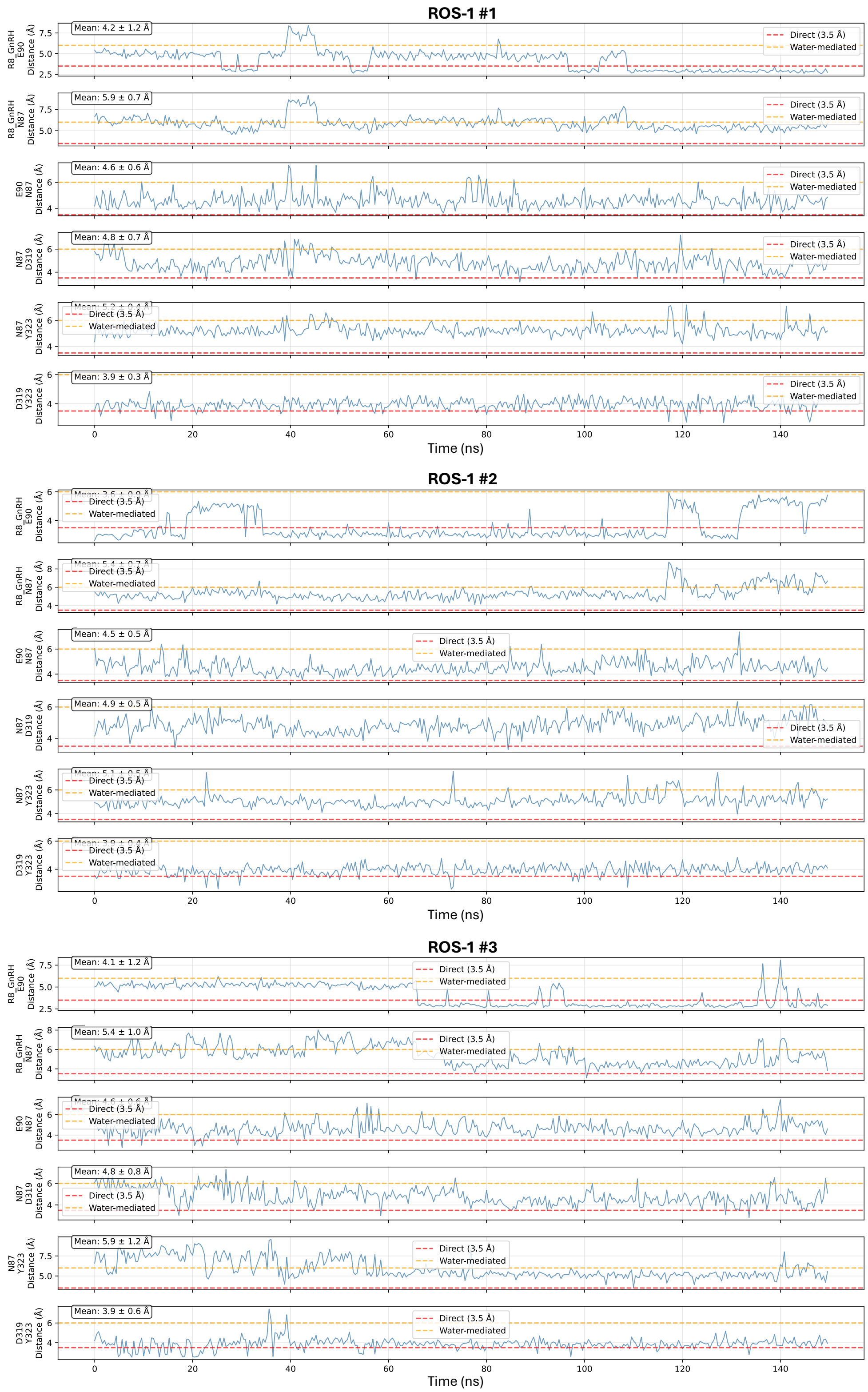


**Figure S9.** Time evolution of water-mediated hydrogen bonds in ROS-1 simulations. Distance trajectories (Å) between selected residue pairs over the last 150 ns for replicas (#1, #2 and #3). Blue lines represent the interaction profile, where the red dashed lines indicate the 3.5 Å cutoff for direct hydrogen bonding and the yellow dashed lines show water-mediated hydrogen bonds.

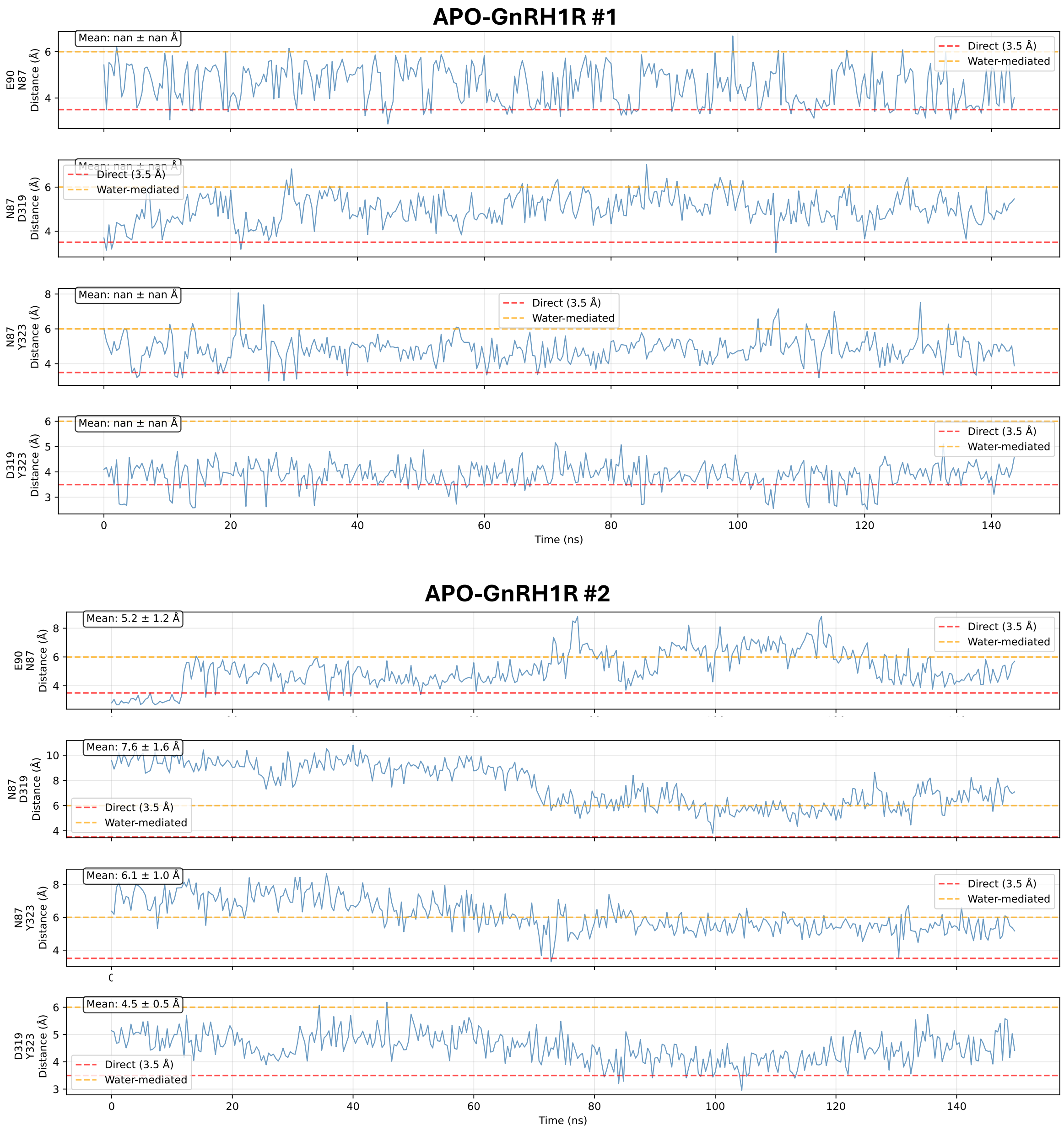


**Figure S10.** Time evolution of water-mediated hydrogen bonds in APO-GnRH1R simulations. Distance trajectories (Å) between selected residue pairs over the last 150 ns for replicas (#1 and #2). Blue lines represent the interaction profile, where the red dashed lines indicate the 3.5 Å cutoff for direct hydrogen bonding and the yellow dashed lines show water-mediated hydrogen bonds.

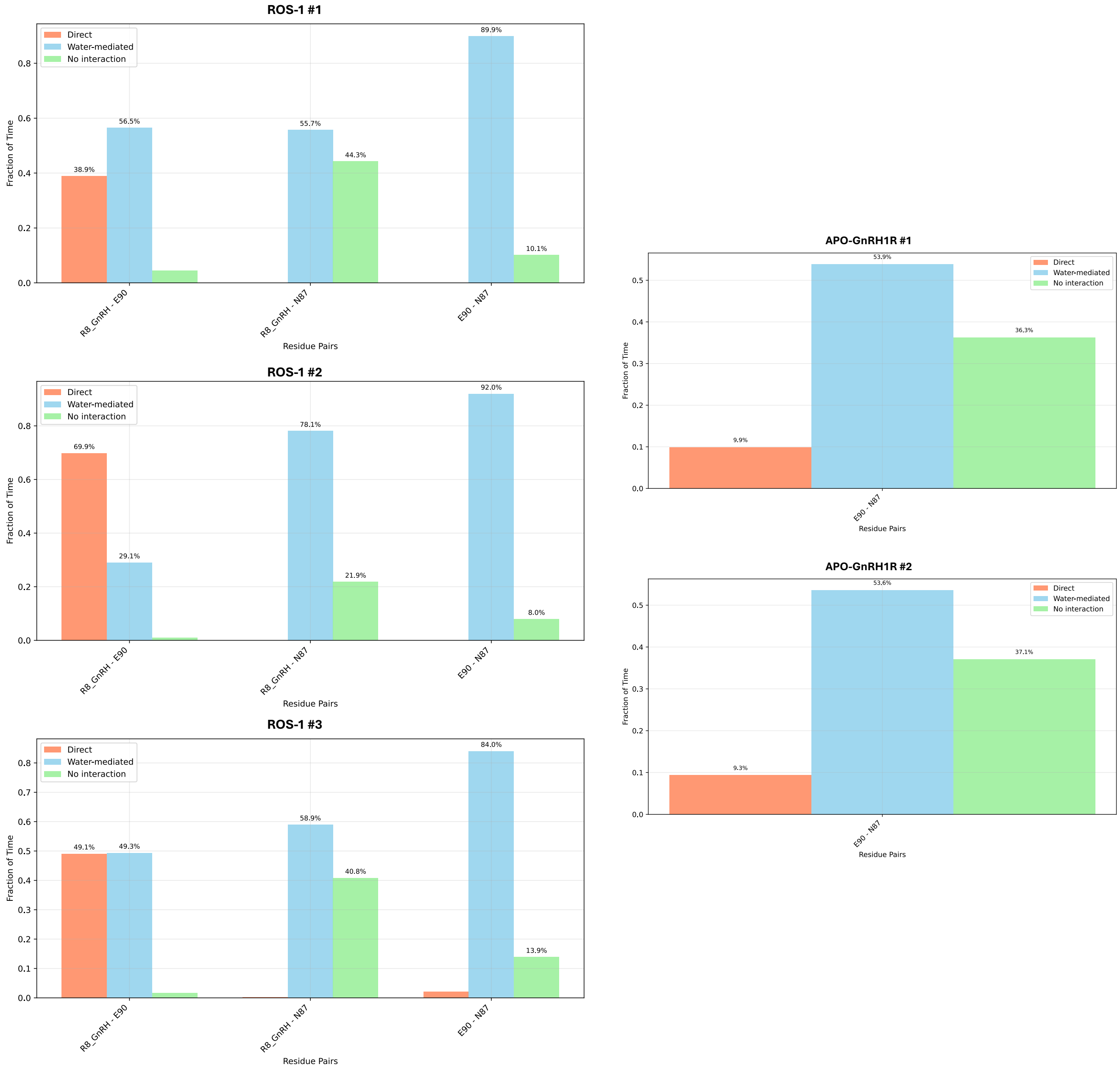


**Figure S11.** Interaction type analysis between key residue pairs in GnRH1R simulations. Stacked bar plots showing the fractional time spent in direct hydrogen bonding (orange), water-mediated interactions (light blue), and no interaction states (green) for selected residue pairs across ROS-1 bound systems (left panels, replicas #1–#3) and APO-GnRH1R systems (right panels, replicas #1–#2). In ROS-1 bound simulations, the R8 (GnRH)–E90 interaction shows predominantly direct contact (38.9–69.8%) with some water-mediation (29.1–56.5%), while R8 (GnRH)–N87 interactions are primarily water-mediated (55.7–78.1%) with significant no-interaction periods (21.9–44.3%). The E90–N87 pair maintains high water-mediated occupancy (83.0–92.0%) across all ROS-1 replicas. In contrast, APO systems show reduced interaction stability, with E90–N87 displaying lower water-mediated occupancy (53.0–57.5%) and increased no-interaction time (36.1–37.1%). The presence of GnRH in ROS-1 appears to stabilise both direct and water-mediated interactions between these functionally important residue pairs.

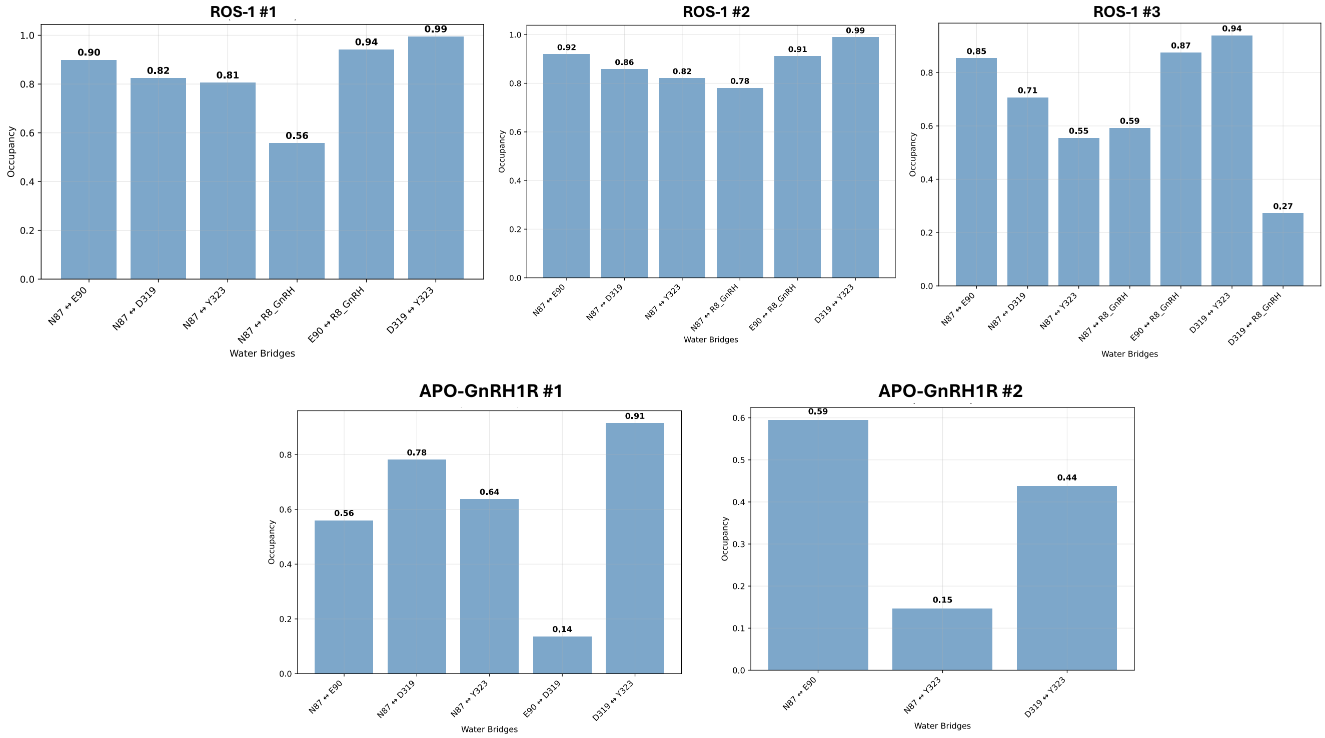


**Figure S12.** Occupancy analysis of water-mediated hydrogen bonds across different GnRH1R simulation systems. Bar plots showing the fractional occupancy of water bridges between key residue pairs in ROS-1 bound simulations (replicas #1, #2, and #3, upper panels) and APO-GnRH1R simulations (replicas #1 and #2, lower panels). Occupancy values represent the fraction of simulation time that water-mediated hydrogen bonds are maintained between the specified residue pairs. In ROS-1 bound systems, water bridges involving N87-E90, N87-D319, N87-Y323, E90-R8 (GnRH), E90-R8 (GnRH), and D319-Y323 show variable occupancies ranging from 0.27 to 0.99 across replicas. The APO systems demonstrate fewer stable water-mediated interactions, with notable bridges between N87-E90 (0.56–0.59), N87-D319 (0.78–0.15), N87-Y323 (0.64–0.44), E90-D319 (0.14), and D319-Y323 (0.91). The presence of the ROS-1 ligand appears to stabilise certain water-mediated networks while disrupting others, suggesting ligand-dependent modulation of the protein hydration shell.

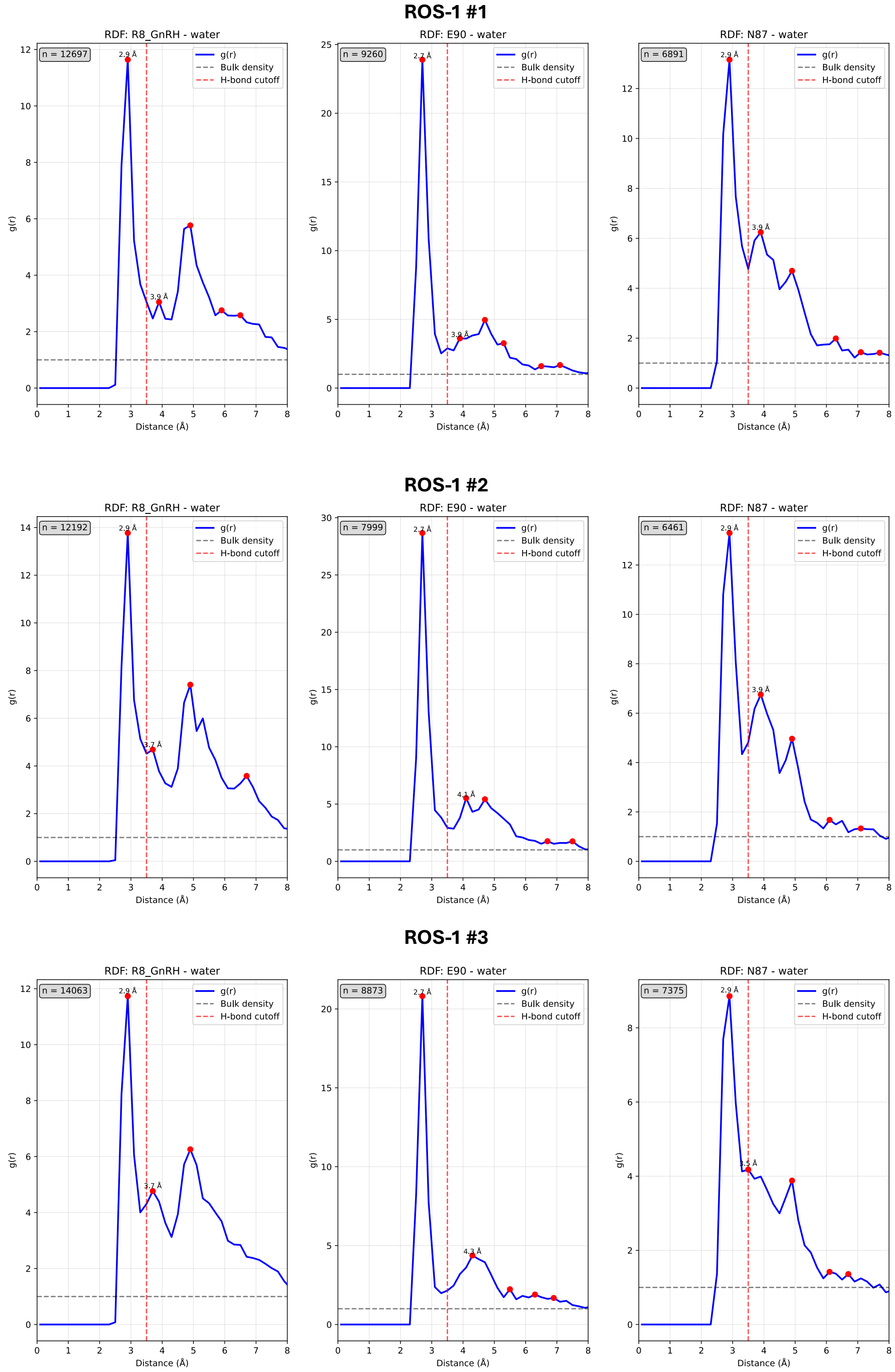


**Figure S13.** Radial distribution functions (RDFs) for water-mediated hydrogen bonds in ROS-1 simulations. RDFs showing the probability density g(r) of water molecules around selected residues E90 (left panels) and N87 (right panels) for replicas #1, #2 and #3). Blue solid lines represent the RDF profiles, gray dashed lines indicate bulk water density, and red dashed lines mark the hydrogen bond cutoff distance. Sharp peaks indicate the first hydration shell, with secondary peaks representing the second coordination sphere. The coordination numbers (n) for each interaction are displayed in the upper left of each panel.

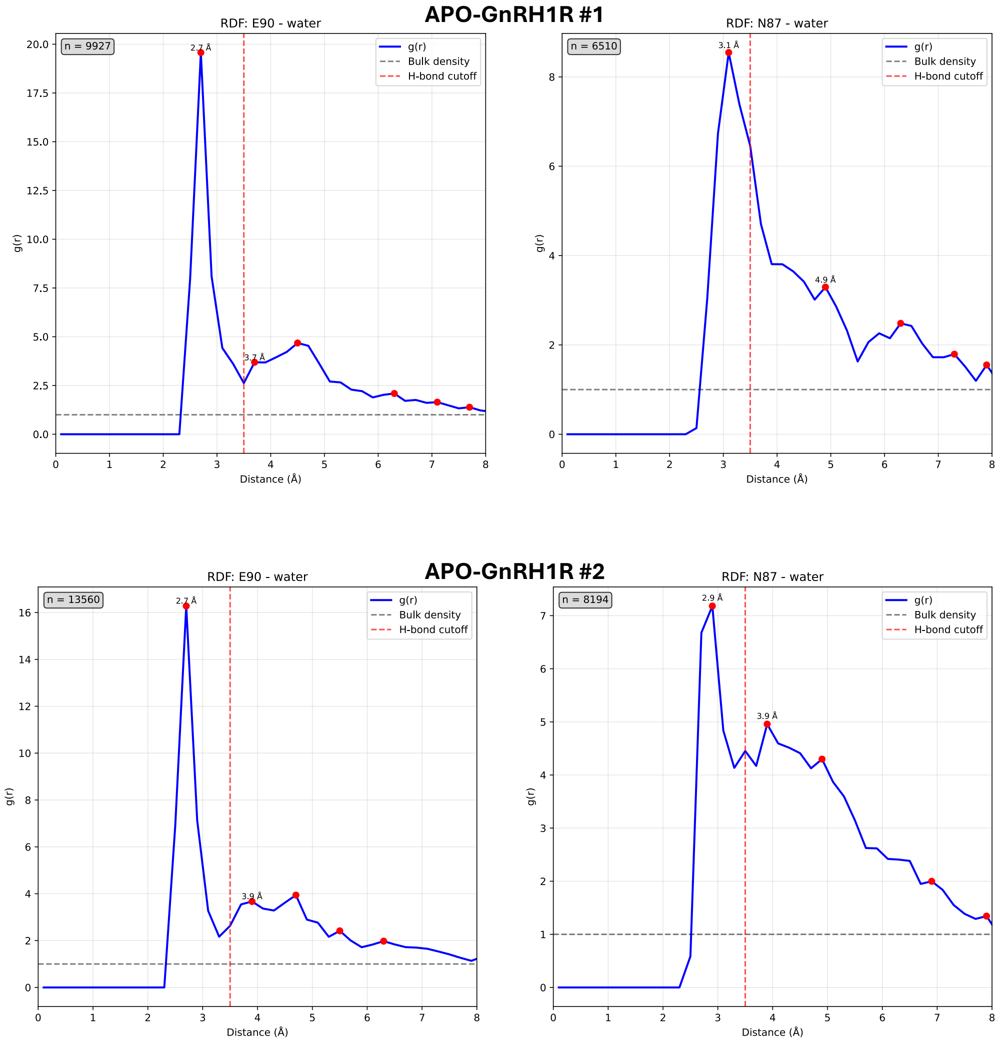


**Figure S14.** RDFs for water-mediated hydrogen bonds in APO-GnRH1R simulations. RDFs showing the probability density g(r) of water molecules around selected residues E90 (left panels) and N87 (right panels) for replicas #1 and #2). Blue solid lines represent the RDF profiles, gray dashed lines indicate bulk water density, and red dashed lines mark the hydrogen bond cutoff distance. Sharp peaks indicate the first hydration shell, with secondary peaks representing the second coordination sphere. The coordination numbers (n) for each interaction are displayed in the upper left of each panel.

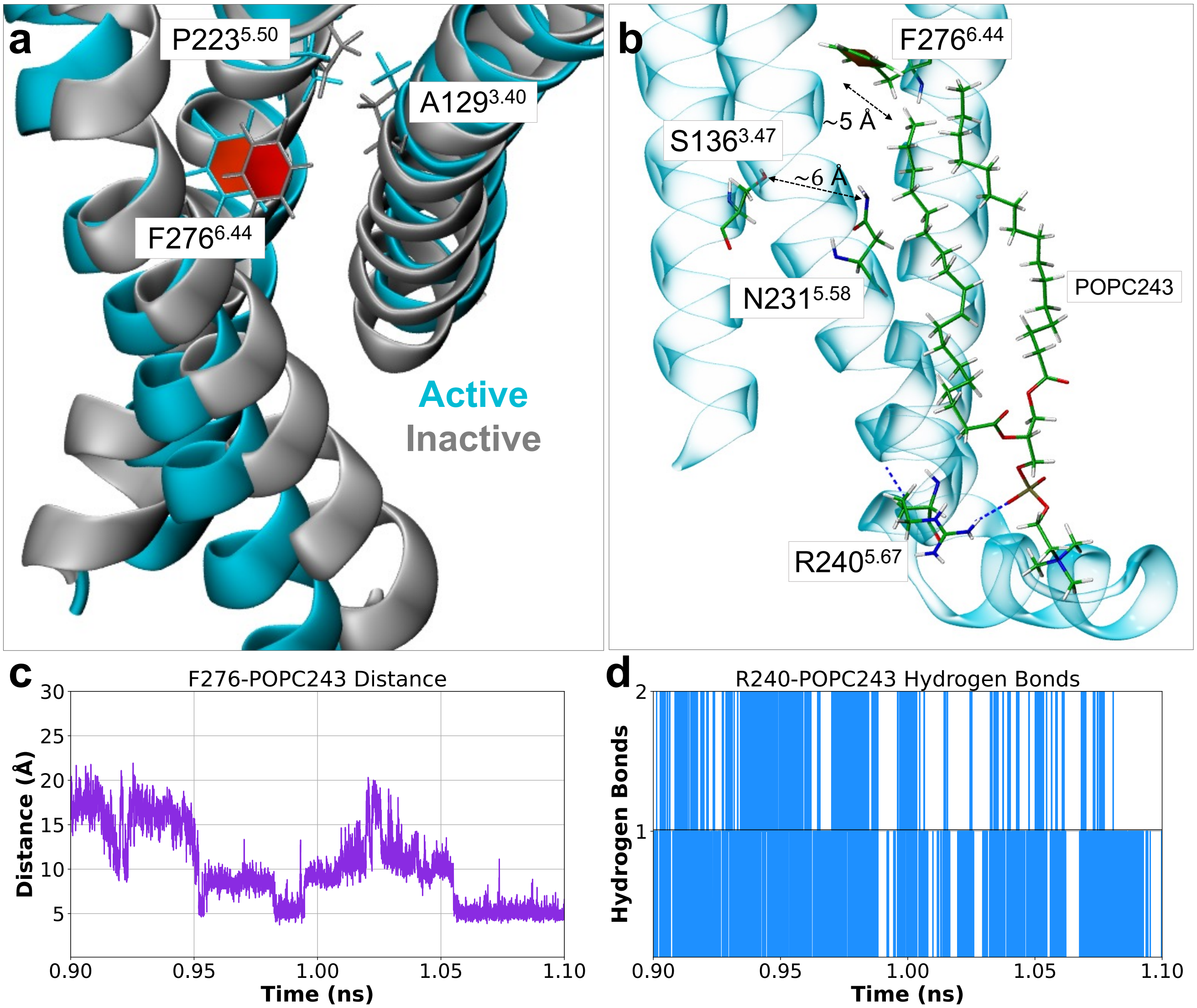


**Figure S15.** a) Comparison of the PAF motif in the inactive Apo-GnRH1R (grey) and active ROS-1 (blue). b) Communication of F276$^{6.44}$ with R240$^{5.67}$ through a common lipid molecule (POPC243). c) Distance between F276$^{6.44}$ and the aryl tail of POPC243. d) Total number of H-bonds formed between R240$^{5.67}$ and POPC243 over time.

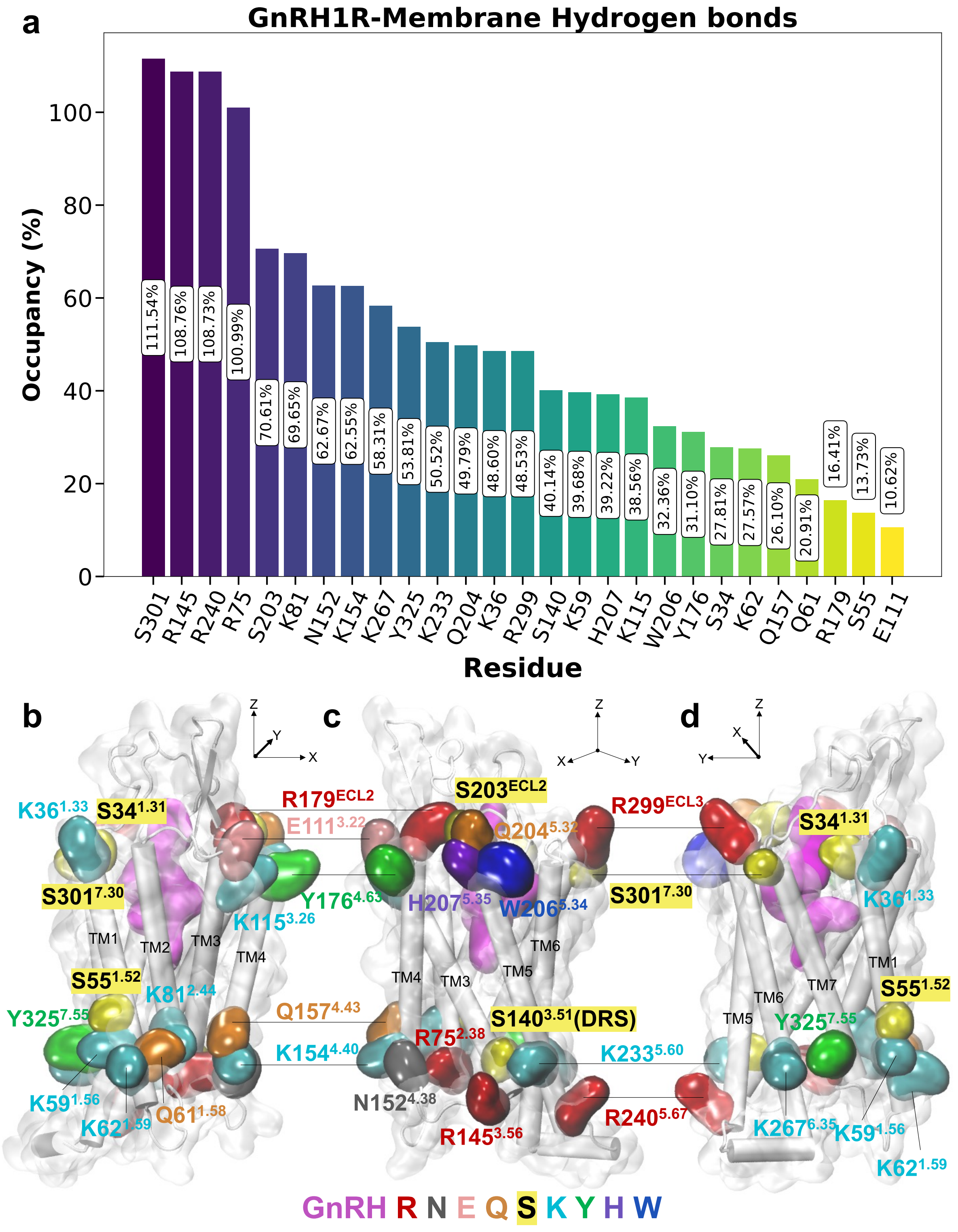


**Figure S16.** Analysis of GnRH1R-Lipid H-bond occupancy in the active ROS-1 System. a) Overall H-bond occupancy between GnRH1R and lipids. b), c), and d) Illustration of GnRH1R residues forming H-bonds with lipids in different orientations around the principal z-axis, represented with surface representation. Note that the occupancy values in graph A exceed 100% because hydrogen bonds between the receptor and lipids are treated as whole residues rather than individual atoms. For example, S301 formed multiple hydrogen bonds with different oxygen atoms of the lipid. To simplify the graph, these individual atom-atom H-bonds were summed, leading to occupancy values greater than 100%.

| Residue | Function (Literature) | This Study |
|---|---|---|
| R38$^{1.35}$ | Interaction with G10 of GnRH (computational study). Forms intrahelical contacts that assist GnRH binding. | R38$^{1.35}$ formed H-bonds with the backbone oxygen of P9 during simulation. However, G10 is a neighbour of P9 and therefore is highly likely to interact also. |
| N87$^{2.50}$ | Receptor functionality: part of the conserved water-mediated network. | N87$^{2.50}$ was found to participate in the water-mediated network involving E90$^{2.53}$-N87$^{2.50}$-DPxxY in the active conformation. In the inactive conformation, due to the binding of a $Na^+$ atom from DPxxY, N87$^{2.50}$ formed one less H-bond with water. |
| D98$^{2.60}$ | Structural or GnRH binding. Interaction with H2 (computational study). Forms conformation-independent ionic locks with K121$^{3.32}$. Adjusts the binding pocket based on the ligand. | D98$^{2.60}$ did not participate in GnRH binding but showed conformation-dependent SB behaviour. The active conformation formed the D98:K121 SB, while the inactive conformation formed the D98:R38 SB. |
| K121$^{3.32}$ | Interacts with non-peptide agonists. Suggested interactions with GnRH residues pG1, H2, and W3 (computational studies). Regulates the conformation of the extracellular side of TM3 by forming a salt bridge with D98$^{2.60}$. The salt bridge is dependent on the nature of the ligand. | Forms a SB with D98$^{2.60}$ in the active conformation. Important for the plasticity of the binding pocket, stabilises the extracellular portions of TM2 and TM3, and forms the outer wall of the binding pocket. |
| M125$^{3.36}$ | Suggested to form the lower wall of the orthosteric pocket in antagonists and blocks access to the toggle switch area (CWxPY). | M125$^{3.36}$ formed H-bonds and vdW with R8 of GnRH. Communicates with the PAF motif through vdW and H-bonds with A129$^{3.40}$, which is located two helical turns below the DRS. |
| Q174$^{4.61}$ | Participates in antagonist binding. | Q174$^{4.61}$ did not participate in GnRH binding. |
| F178$^{4.65}$ | Participates in antagonist binding. | F178$^{4.65}$ did not participate in GnRH binding. |
| W280$^{6.48}$ | Part of the toggle switch (CWxPY), this residue has been shown to directly contact ligands in various GPCRs. Mutation abolishes signalling response. Suggested to interact with W3 through $\pi-\pi$ interactions. | W280$^{6.48}$ forms cation-$\pi$ interactions with R8 and $\pi-\pi$ interactions with F276$^{6.44}$, F272$^{6.40}$, and Y323$^{7.53}$ in the active conformation. This network is closed in the inactive conformation. |
| Y283$^{6.51}$ | Belongs to the CWxPY motif. Crucial for ligand binding and activation. Suggested to assist in GnRH binding between W3 and Y283$^{6.51}$ upon disruption of the Y283$^{6.51}$-F309$^{7.38}$ intrahelical contact (computational studies). | Forms $\pi-\pi$ interactions with Y5 and F309$^{309}$. Belongs to the group of $\pi-\pi$ interactions consisting of W3, Y283$^{6.51}$, Y290$^{6.58}$, H306$^{7.35}$, and F309$^{7.38}$. |
| L286$^{6.54}$ | Interacts with antagonists. | L286$^{6.54}$ formed H-bonds and vdW interactions with the alcohol group of Y5. Additionally, it formed H-bonds and vdW interactions with Y290$^{6.58}$ and Y284$^{6.53}$, respectively. |
| Y290$^{6.58}$ | Suggested interaction with Y5 of GnRH. | Y290$^{6.58}$ was found to form $\pi-\pi$ interactions with W3 and Y5, and was part of the $\pi-\pi$ network including Y283$^{6.51}$, Y290$^{6.58}$, H306$^{7.35}$, and F309$^{7}$. |
| D302$^{7.31}$ | Suggested interaction with R8 through a salt bridge. | D302$^{7.31}$ did not participate in GnRH binding. It supported the interaction of H306$^{7.35}$ with W3 and F309$^{7.38}$ through vdW and H-bonds with H306$^{7.35}$. |
| H306$^{7.35}$ | Suggested to form intrahelical contact with F308$^{7.37}$, which consequently forms $\pi-\pi$ interactions with W3 of GnRH. | H306$^{7.35}$ was found to interact with W3 of GnRH through $\pi-\pi$ interactions. The orientation of F308$^{7.37}$ was towards the membrane environment, where it could assist the stability of the binding pocket through hydrophobic interactions with the membrane. |

| Residue | Function (Literature) | This Study |
|---|---|---|
| NPxxY | Participates in the conformation-independent conserved interhelical network in the water-mediated polar network and forms conformation-specific interhelical interactions. | In the inactive conformation, Y323$^{7.53}$ of DPxxY interacts with TM1 residues F56$^{1.53}$ and W63$^{1.60}$. In the active conformation, the pathway to CWxPY is open, where Y323$^{7.53}$ communicates with W280$^{6.48}$/R8 through F272$^{6.40}$ and F276$^{6.44}$ of the PAF motif. D319$^{7.49}$ of DPxxY acts as a sodium binding residue in the inactive conformation and participates in the water-mediated network between GnRH/CWxPY and E90$^{2.53}$/N87$^{2.50}$ and DPxxY. |
| DRY | G-protein interaction site. R3.50 of the DRY motif forms an ionic lock with E6.30 in numerous GPCRs. An increase of the distance between these residues (>5 Å) commonly indicates the conformational state of the GPCR. The corresponding residues in GnRH1R are R139$^{3.50}$ and T265$^{6.33}$, which form a polar interaction in the inactive conformation that stabilises the distance between TM3 and TM6 at 7.5 Å. | In the active conformation, the distance between R139$^{3.50}$ and T265$^{6.33}$ increases to 13 Å. The DRS and PAF motifs interact through M125$^{3.36}$, which interacts directly with R8. S140$^{3.51}$ of the DRS interacts with lipids through H-bonds. D138$^{3.49}$ of the DRS forms a SB with R75$^{2.38}$, which also interacts with a lipid through H-bonds. |
| F276$^{6.44}$ and N231$^{5.58}$ | N231$^{5.58}$ is associated with receptor activation. | F276$^{6.44}$ of the PAF rotates towards TM5 and membrane environment in the active conformation, where it forms vdW interactions with a lipid molecule. The same lipid molecule acts as a mediator as it also interacts with N231$^{5.58}$. The distance between N231$^{5.58}$ and S136$^{3.47}$ in the inactive conformation was 4 Å, while it increased to 6 Å in the active conformation. Additionally, F276$^{6.44}$ was observed within 5 Å of a lipid tail, which also interacts with R240$^{5.67}$ through H-bonds in the active conformation. |
| F272$^{6.40}$ | In most GPCRs, the residue in position 6.40 is a short hydrophobic amino acid. The GnRH1R has a phenylalanine in this position, and it is highly conserved in tailless mammalian GnRH receptors. F272$^{6.40}$ has been found important for activation, but its exact function was not discovered. | Mediates interactions between Y323$^{7.53}$ (DPxxY motif), F276$^{6.44}$ (PAF motif), and subsequently W280$^{6.48}$ (CWxPY motif) through $\pi-\pi$ interactions in the active conformation. |

**Table S3.** Comparative summary between the GPCR/GnRH1R characteristics mentioned in the literature and those found in this study for the GnRH1R.

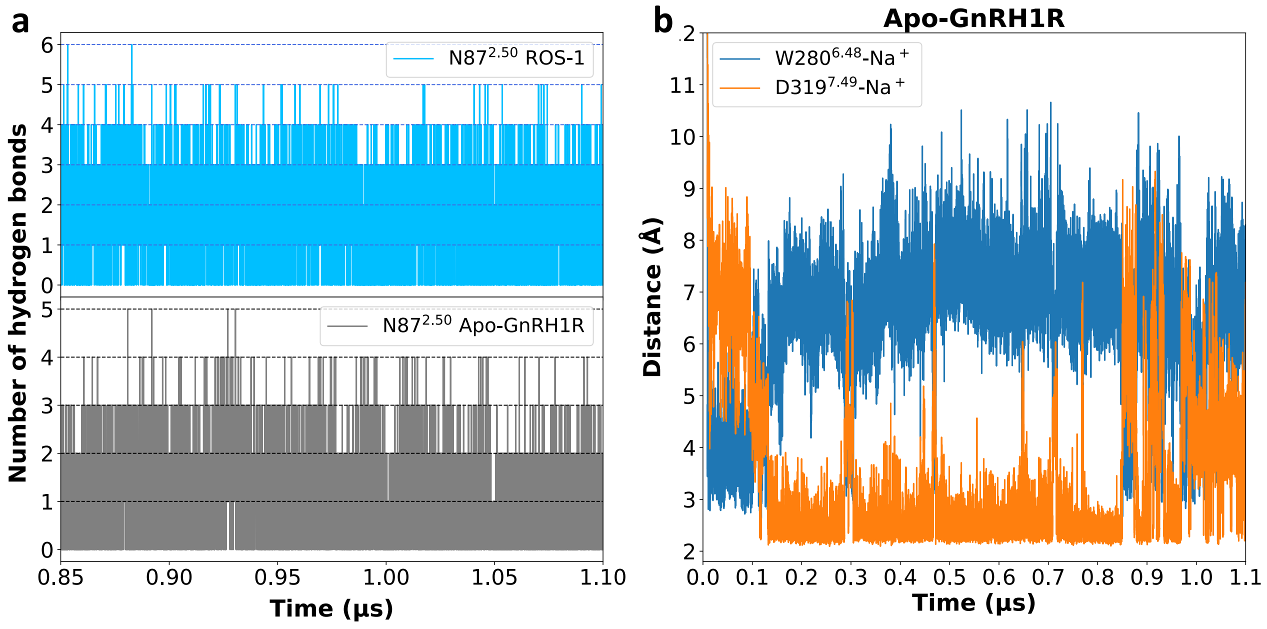


**Figure S17.** a) Hydrogen bonds between N87$^{2.50}$ and water molecules in the ROS-1 and Apo-GnRH1R simulations. b) Distance of W280$^{6.48}$ and D319$^{7.49}$ with the sodium atom.

## Rosetta-Docking candidate selection protocol

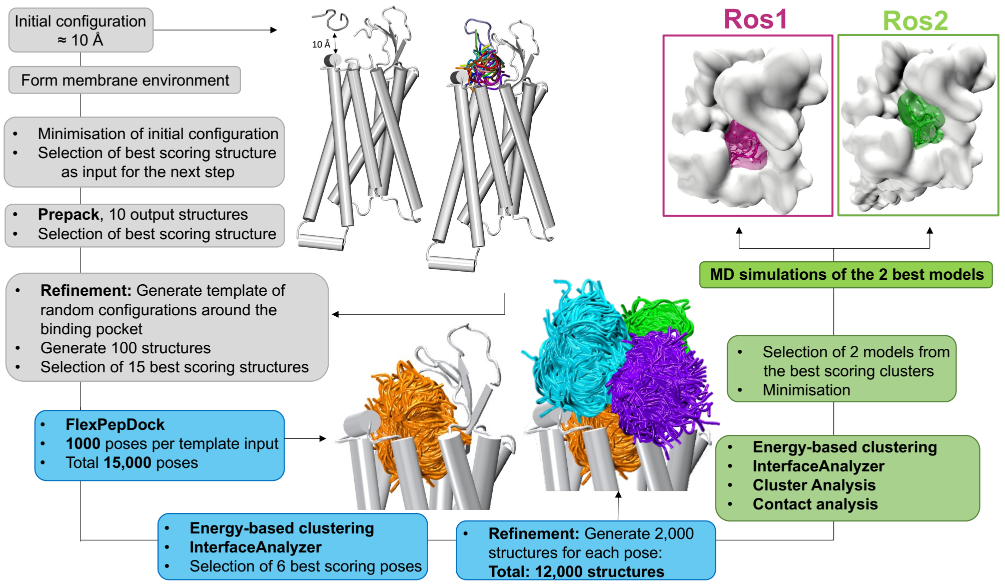


**Figure S18.** GnRH to GnRH1R docking pipeline: Structure preparation and template formation (grey); Initial docking and final refinement structure selection (blue); Binding mode selection *via* cluster, contact, and hydrogen bond analysis (green). Optimal binding poses meeting criteria were chosen for MD simulations.

The analysis began with 12,000 final docking poses that underwent energy-based clustering, resulting in a total of 84 clusters, with populations ranging from 2,022 to 47 structures. Only clusters with populations greater than 50 were selected for subsequent analysis. This led to the identification of 17 clusters, with populations varying from 2,022 to 52. Further analysis involved excluding structures with a Total Score greater than -620 REU and a $\Delta G$ Binding greater than 0 REU. Violin plots

were constructed for the Total Score, $\Delta G$ Binding, and $\Delta SASA$ of the interface for these clusters (Figure S19, Table S4).

Clustering was conducted based on Total Score metrics, where clusters comprised conformations sharing similar RMSD values. Due to the stochastic nature of Rosetta, it is possible for a cluster to include conformations that are structurally similar but exhibit a wide range of Total Scores. In such instances, priority is given to clusters with lower Total Scores, as they are more indicative of physically plausible states. Higher-energy clusters may be trapped in local minima, which a physical receptor would typically overcome easily.

In the subsequent elimination phase, clusters with populations fewer than 1,000 structures were excluded from further consideration. Consequently, clusters 2, 4, 9, 1, and 5 were taken into account, with populations of 1,761, 1,523, 1,378, 1,129, and 1,083 respectively (Figure S19, Table S4). However, despite its significant size, Cluster 9's low $\Delta G$ Binding values did not lead to a corresponding reduction in Total Score (Figure S19). Cluster 9 displayed the highest Total Score values at approximately -650 REU, while the remaining clusters had values closer to -680 REU. Given that all clusters exhibited similar lower $\Delta G$ Binding values, it would be expected that favourable conformations within Cluster 9 would also have a lower Total Score. This inconsistency suggests that the conformations within Cluster 9 may not represent energetically favourable states despite their abundance. Therefore, Cluster 9 was deemed unreliable for containing biologically relevant conformations and was excluded from further analysis. The final clusters: 2, 4, 1, 5, were then subjected to statistical analysis.

**Table S4.** Cluster population before and after applying elimination criteria. Structures with Total Score values > -620 REU and $\Delta G$ Binding > 0 were eliminated.

| Cluster name | Population before elimination | Population after elimination | Structures eliminated |
|---|---|---|---|
| 2 | 2022 | 1761 | 261 |
| 4 | 1809 | 1523 | 286 |
| 9 | 1787 | 1378 | 409 |
| 1 | 1763 | 1129 | 634 |
| 5 | 1528 | 1083 | 445 |
| 3 | 1573 | 929 | 644 |
| 8 | 507 | 295 | 212 |
| 7 | 190 | 91 | 99 |
| 11 | 109 | 73 | 36 |
| 6 | 81 | 50 | 31 |
| 10 | 73 | 42 | 31 |
| 18 | 63 | 38 | 25 |
| 14 | 66 | 34 | 32 |
| 19 | 107 | 31 | 76 |
| 16 | 67 | 30 | 37 |
| 21 | 54 | 21 | 33 |
| 17 | 52 | 15 | 37 |

Prior to conducting statistical analysis on the chosen clusters, evaluation of the data distributions was deemed necessary for the application of appropriate statistical tests. As a result, the Kolmogorov-Smirnov (K-S) statistical test, in conjunction with histograms and Quantile-Quantile (Q-Q) plots of each metric, was carried out to gain an understanding of the data distributions for all selected clusters (Figure S20). These tests offer insights into whether the data deviate or adhere to a normal Gaussian distribution. Histograms provide a visual representation of the frequency distribution of each variable and the overall shape of the distributions. Q-Q plots allow for a comparison of the distribution of metrics with a theoretical normal distribution.

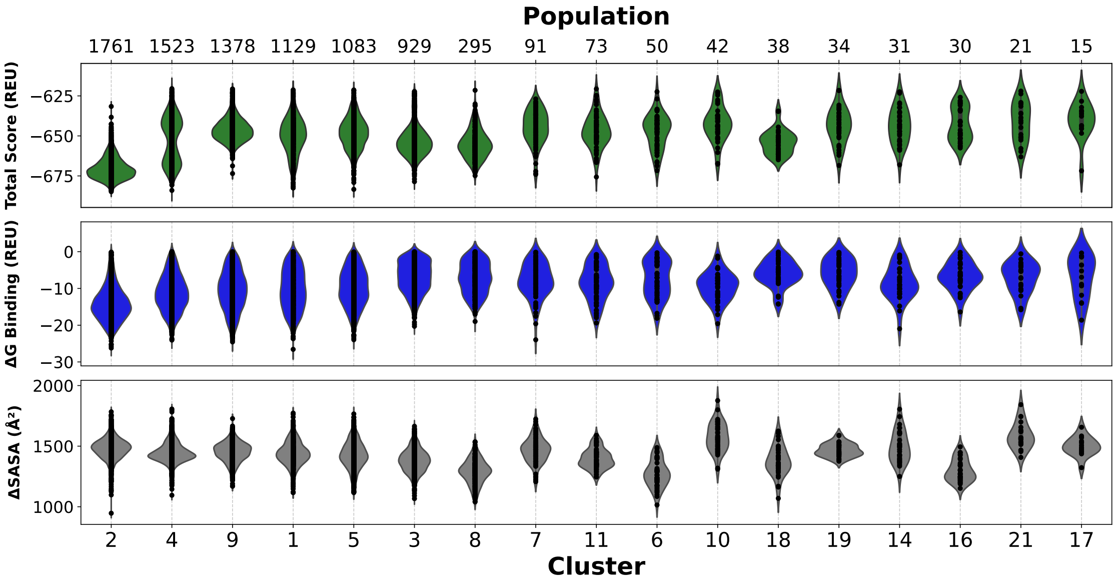


**Figure S19.** Energy metrics of the 17 most populated clusters: Total Rosetta score (REU) in green, $\Delta G$ Binding (REU) in blue, and $\Delta$SASA (Å$^2$) of the binding interface in grey. These metrics are displayed as distributions in violin plots. Cluster identifiers are shown on the lower x-axis, while cluster populations are indicated on the upper x-axis.

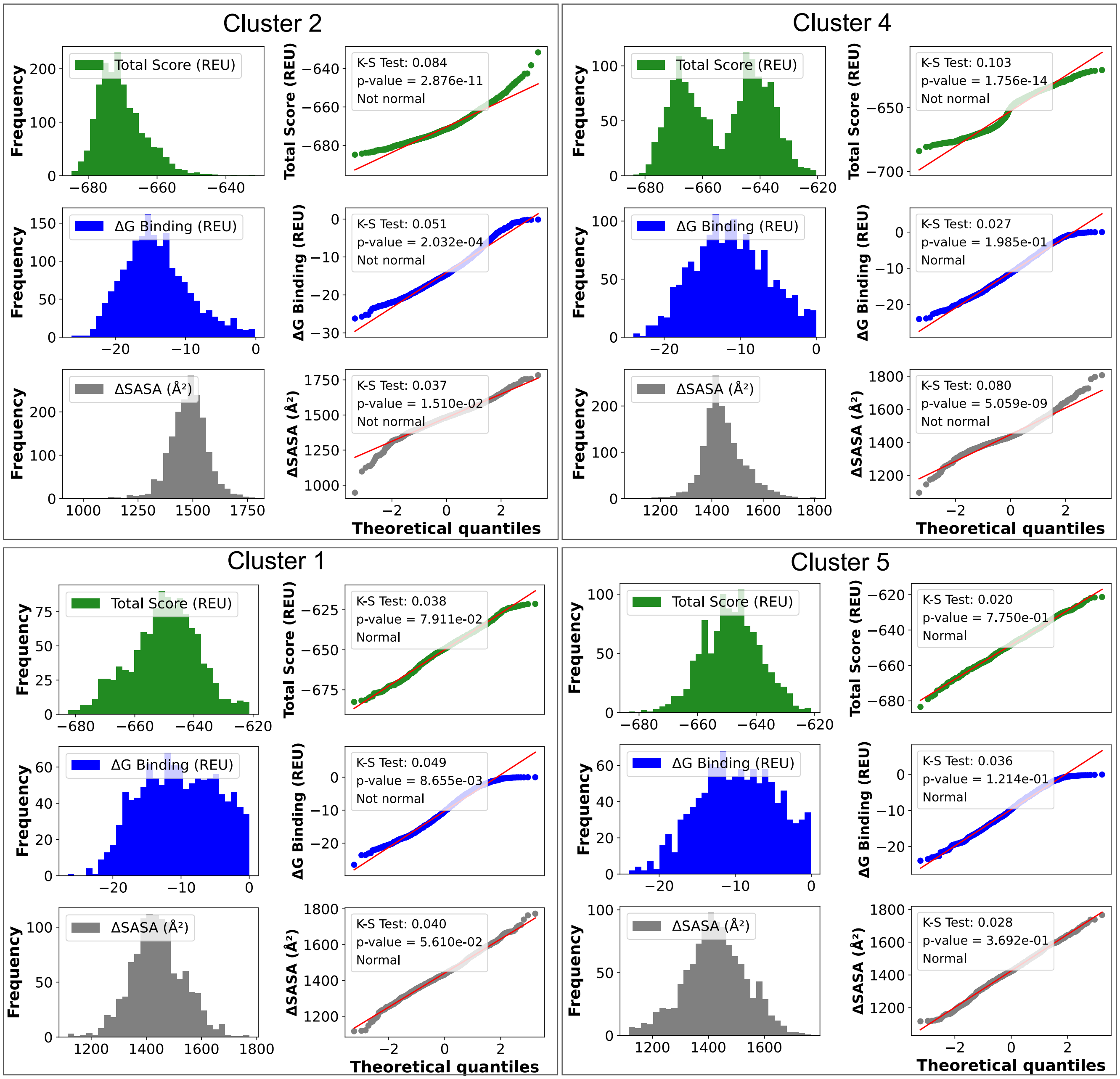


**Figure S20.** Histograms (left) and Q-Q plots (right) for Total Score (REU) in green, Δ$G$ Binding (REU) in blue, and ΔSASA (Å$^2$) in grey across Clusters 2, 4, 1, and 5. The legend includes p-values against a theoretical Gaussian distribution (red line) along with the Kolmogorov-Smirnov coefficient. Results from normality tests are also displayed within each legend.

**Table S5.** K-S test results for all tested metrics in Clusters 2, 4, 1, and 5.

| Cluster | Total Score (REU) | Δ$G$ Binding | ΔASA (Å$^2$) |
|---|---|---|---|
| Cluster 2 | Not normal | Not normal | Not normal |
| Cluster 4 | Not normal | Normal | Not normal |
| Cluster 1 | Normal | Not normal | Normal |
| Cluster 5 | Normal | Normal | Normal |

The K-S test determines whether the distribution follows normality or not as follows:

$$D_n = \sup_x |F_n(x) - F(x)| \quad (1)$$

Where $D_n$ represents the K-S statistic, $F_n(x)$ is the empirical distribution function of the sample, and $F(x)$ is the cumulative distribution function of the compared distribution (in this case, the normal distribution). The K-S statistic, along with the p-value, is employed to assess the hypothesis that the data in each case conform to a normal distribution. In this context, if $p > 0.05$, then the data is considered to follow a normal distribution; otherwise, it is considered not to follow a normal distribution. The results of this analysis offer insights into whether parametric or nonparametric statistical tests can be applied to the cluster metrics, as parametric tests can only be applied to normal distributions. The p-value of the K-S test was computed as an approximation for large data sets in the following manner:

$$p \approx 1 - 2\sum_{k=1}^{\infty} (-1)^{k-1} e^{-2k^2 D_n^2} \quad (2)$$

Where 1 represents the initial point of the calculation and signifies the total probability prior to subtracting the calculated probability of the observed test statistic. The term $(-1)^k = \pm 1$ ensures the convergence of the series for both positive and negative values, corresponding to even and odd $k$ values, respectively. The K-S statistic, denoted as $D$, stands for the maximum distance between the empirical cumulative distribution function of the sample and the theoretical distribution. Multiplying $D$ by the sample size, $n$, holds significance as larger sample sizes enhance the ability of the test to detect disparities between the sample distribution and the theoretical distribution. As the K-S test demonstrates that half of the metric distributions conform to normality, while the other half deviate from normality (Figure S20, Table S5), the choice of non-parametric statistical tests for further analysis was made. This preference arises from the inherent flexibility of non-parametric approaches in accommodating mixed distribution types, their robustness against data peculiarities such as skewness and outliers, and their independence from the stringent assumptions that underlie parametric tests. Especially in the upcoming cluster statistical analysis, the objective is to identify and categorise inherent structures within multidimensional data. This enhances the validity and generalisability of the findings and embodies a methodologically sound approach in situations characterised by heterogeneous data distributions.

The selected clusters were presented in the form of violin plots, which offer a valuable means of visualising distinct behavioural patterns for each evaluated metric. Non-parametric double-sided Mann-Whitney U tests were employed to evaluate each pairwise combination of the four clusters (Figure S21). The null hypothesis assumed was no significant difference between the distributions of the two clusters in comparison. The test involves ranking all the data points from both groups together and then analysing the sum of ranks in each group. A significant result (p-value < 0.05) indicates that the likelihood of observing the difference in ranks by random chance is low, suggesting a genuine difference of the data distributions. Conversely, p-values > 0.05 suggest similar values for the groups. The U values of the Mann-Whitney double-sided test are calculated as follows:

$$U_X = n_X \cdot n_Y + \frac{n_X(n_X+1)}{2} - R_X \quad (3)$$

Where $U_X$ represents the Mann-Whitney U statistic for sample X, $n_X$ is the number of observations in sample X, $n_Y$ is the number of observations in sample Y, and $R_X$ is the sum of the ranks of the observations in sample X when all observations from both samples X and Y are ranked together. The product $n_X \cdot n_Y$ represents the total number of possible pairings between an observation in sample X and an observation in sample Y. Each pairing contributes to the overall ranking comparison. The term $\frac{n_X(n_X+1)}{2}$ calculates the sum of the ranks that would be assigned to sample X based on the formula for the sum of the first n natural numbers, which is $\frac{n_X(n_X+1)}{2}$. The subtraction of $R_X$ from this term accounts for the actual ranks obtained by sample X. Essentially, U quantifies the extent of rank dominance of one sample over the other. A small U value indicates that most observations in sample X tend to have lower ranks (are smaller) compared to those in sample Y. Conversely, a large U value suggests that observations in sample X tend to have higher ranks (are larger). The Mann-Whitney U test employs this statistic to determine whether there is a statistically significant difference between the two independent samples in terms of their rank ordering. The $U_Y$ statistic is calculated separately in a similar way using the modified version of (Equation 3) for sample Y and the smaller U value is used for the p-value calculation. In cases with large sample sizes, the U statistic approximates a normal distribution, and the z-score is computed as follows:

$$z = \frac{U - \mu_U}{\sigma_U} \quad (4)$$

where, $\mu_U = n_1 n_2 / 2$ is the mean and $\sigma_U = \sqrt{n_1 n_2 (n_1 + n_2 + 1)/12}$ is the standard deviation of the U distribution.

The p-value is computed based on the minimum U statistic and it indicates whether the observed difference in rank ordering between the two samples is statistically significant. For a double-sided test, the p-value is computed using the z-score as follows:

$$p = 2 \cdot P(Z > |z|) \tag{5}$$

This calculation places particular emphasis on both tails of the distribution by determining the probability of a z-score greater than the absolute value of the calculated z-score. The $P(Z > |z|)$ part of the equation involves the standard normal random distribution (Z) and represents the probability (P) that a standard normal random variable Z is greater or equal to the absolute value of the test statistic |z|. The multiplication by 2 is performed to facilitate the double-sided test.

Furthermore, to account for multiple comparisons, the Bonferroni correction was applied to adjust the p-values obtained from the pairwise Mann-Whitney U tests. The Bonferroni correction is a method used to control the family-wise error rate (FWER), which represents the probability of making at least one Type I error (false positive) among all the conducted tests. The Bonferroni correction is calculated as follows:

$$p - value(Bonferroni) = p - value \cdot N \tag{6}$$

where N is the number of comparison and is calculated as:

$$N = \frac{N_c \cdot (N_c - 1)}{2} \tag{7}$$

where $N_c$ is the number of clusters being compared, and the division by 2 is used due to the double-sided comparison. This correction is employed to reduce the likelihood of obtaining a significant result by chance when conducting multiple tests. It is essential to note that while p-values indicate the presence or absence of an effect, they do not quantify the size of the effect. Therefore, additional statistical measures, such as Effect Size (ES), are reported to measure the magnitude of differences observed between the clusters. In the context of the Mann-Whitney U test, ES is a metric that calculates the magnitude of difference between two groups. It is computed using the formula:

$$ES = 1 - \frac{2U}{n_1 n_2} \tag{8}$$

where U is the Mann-Whitney U statistic, and $n_1$ and $n_2$ are the sample sizes of the two groups. This ES value signifies the extent of overlap between the distributions of the two groups, offering a standardised measure of the magnitude of the difference. An ES value nearing 0 indicates a substantial overlap, suggesting a small or negligible effect (similar distributions). Conversely, a value closer to 1 implies less overlap, signifying a larger effect (different distributions).

In this analysis, the Total Score, $\Delta G$ Binding, and $\Delta SASA$ of the interface among the most populated clusters were compared. The objective of this analysis is to uncover similarities and differences in these metrics among the clusters, leading to a deeper understanding of their representative binding modes. Specifically, all clusters exhibited statistically significant differences in their Total Score values, as indicated by p-values < 0.05 (Figure S21). The most significant differences were observed in the comparisons of Cluster 2 with the three other clusters, whereas smaller differences were observed between pairs of Clusters 4, 1, and 5. This disparity is also evident in the significant difference in the median Total Score value, with Cluster 2 at approximately -670 REU and Clusters 4, 1, and 5 at approximately -650 REU. This difference is also reflected by the large ES values. Conversely, although statistically different, Clusters 4, 1, and 5 displayed more subtle differences in the Total Score, with small or moderate ES values. Intriguingly, Cluster 4 was composed of two distinct groups of energy ‘subclusters’, yielding Total Scores of approximately -640 and approximately -665 REU (Figure S21).

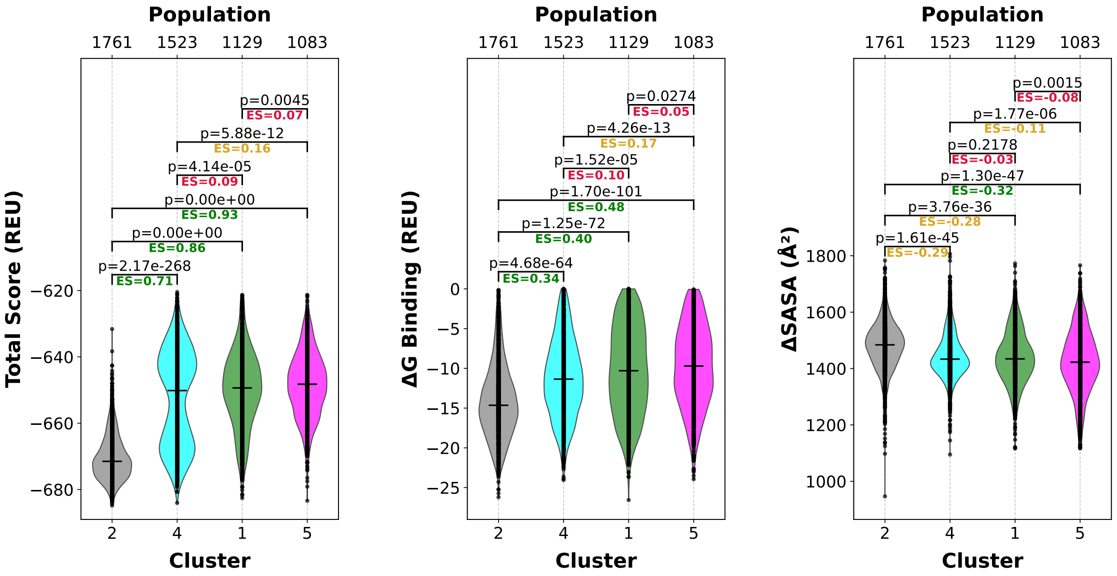


**Figure S21.** Chosen Clusters for in-depth cluster analysis: a) Total Score (REU), b) $\Delta G$ of binding (REU), and c) $\Delta$SASA buried at the interface of selected clusters. Population of each cluster post-elimination is shown on the upper x-axis. P-values calculated using double-sided Mann-Whitney U test with Bonferroni correction. ES is indicated as large (green), moderate (yellow), and small (red) effects.

In terms of $\Delta G$ Binding, Cluster 2 exhibits a significant median value difference of approximately -15 REU, while Clusters 4, 1, and 5 have values ranging from -12 to -10 REU. All clusters exhibit significant differences in terms of $\Delta G$ Binding except for the comparison of Clusters 1 and 5 ($p > 0.05$) (Figure S21). This suggests that Clusters 1 and 5 include similar binding poses in terms of energy. However, as the $\Delta SASA$ comparison is significantly different ($p < 0.05$), these clusters must exhibit significant differences in their conformations. The decrease in the $\Delta SASA$ of Cluster 5 compared to Cluster 1, indicated by the small negative decrease (-0.08) in ES, suggests that conformations within Cluster 5 induce a stronger conformational change than those in Cluster 1. However, both clusters are similar in terms of $\Delta SASA$ and $\Delta G$ Binding compared to the other compared clusters. Furthermore, small and moderate ES values between Clusters 4-1 and 4-5 indicate that the dual population of Cluster 4 (as indicated in the Total Score) yields conformations with similar $\Delta G$ Binding to either Cluster 1 or 5, while more similar to those of Cluster 1, as indicated by the small values of their $\Delta SASA$ ES (-0.03) (Figure S11).

It is intriguing that despite the extremely low values of Cluster 2 in both Total Score and $\Delta G$ Binding, the median $\Delta SASA$ values are increased compared to the rest of the clusters. This anomaly may serve as an indication that GnRH conformations within Cluster 2 result in highly favourable binding energies due to possible interference with the N-terminus, possibly indicating a false-positive docking pool [1]. Since a decrease in $\Delta SASA$ values typically indicates a significant conformational change, the overall increase observed in Cluster 2 may suggest the existence of biologically irrelevant conformations. The lowest $\Delta SASA$ values are observed in conformations within Cluster 5, followed by Clusters 1 and 4. Further analysis of the specific contacts between GnRH and GnRH1R are essential to gain a deeper understanding of the nature of these differences (Figure S11).

Contact analysis was conducted for the selected clusters to extract specific characteristics of the various binding modes. In this analysis, only structures that exhibited at least one contact between GnRH and GnRH1R were considered (Figure S22). Contacts were identified based on a 5 Å cutoff distance between $C\beta$ carbons of the peptide-receptor complex ($C\alpha$ for glycine).

This elimination criterion led to a substantial reduction in the cluster populations, as numerous structures did not display any contacts and were thus excluded from further consideration (Table S6).

**Table S6.** Cluster population before and after the second elimination round, applying criteria requiring at least one contact.

| Cluster number | Population before 2$^{nd}$ elimination | Population after 2$^{nd}$ elimination | Structures eliminated |
|---|---|---|---|
| 2 | 1761 | 10 | 1751 |
| 4 | 1523 | 1093 | 430 |
| 1 | 1129 | 838 | 291 |
| 5 | 1083 | 947 | 136 |

It is noteworthy that the majority of population within Cluster 2 was excluded based on the second elimination criterion (Figure S22a). This further supports previous indications that the binding modes of Cluster 2 may be part of the false-positive dockings. As only 10 structures of this cluster formed at least one contact with the receptor, and notably, only one pose showed two important contacts with residues L23 and Y290$^{6.58}$, this cluster was deemed unsuitable for further analysis.

Similarly, despite the high population numbers in Cluster 4, only the important contact Y290$^{6.58}$-GnRH was identified (Figure S22b). In contrast, Clusters 1 and 5 exhibit a substantial number of important contacts and a significant population (Figure S22c, d). This implies that the docking configurations within these clusters have a strong likelihood of including the close-to-native GnRH and GnRH1R.

As clusters 2 and 4 did not present high numbers or important contacts, hydrogen bond analysis was conducted only for clusters 1 and 5 (Figure S23). The aim of the contact and H-bond analysis was to firstly eliminate poses that do not present any meaningful contacts, and additionally perform an in-depth analysis of the binding interface of the clusters. Special emphasis is given to contacts and H-bonds formed between GnRH and the GnRH1R binding site.

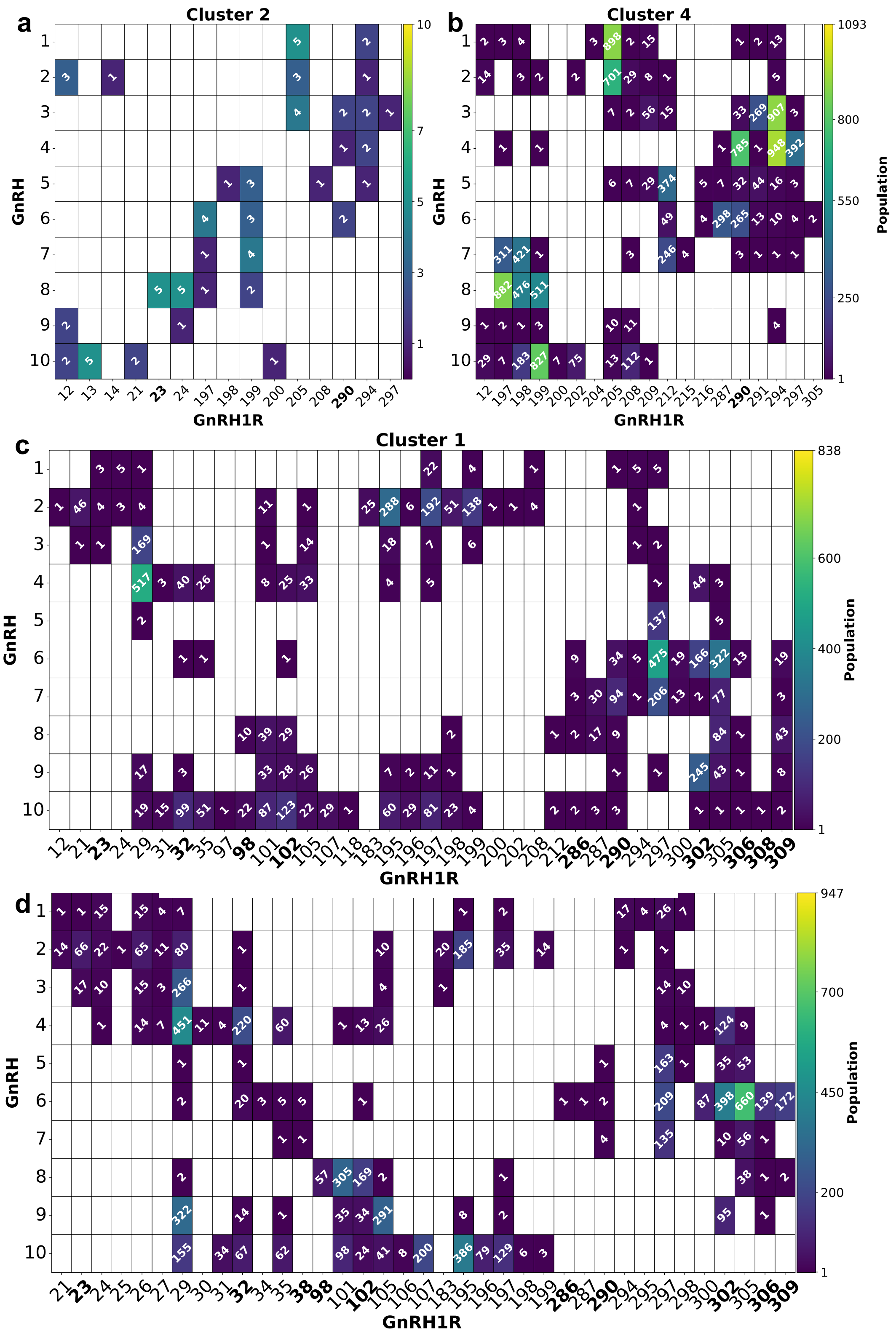


**Figure S22.** Contact analysis of Clusters 2 (a), 4 (b), 1 (c), and 5 (d) in the form of heatmaps. The Population bar represents the number of structures that display the specific contact in a colour-coded manner: high (yellow) and low (blue) Population. In bold font, experimentally proven important GnRH-interacting residues are highlighted.

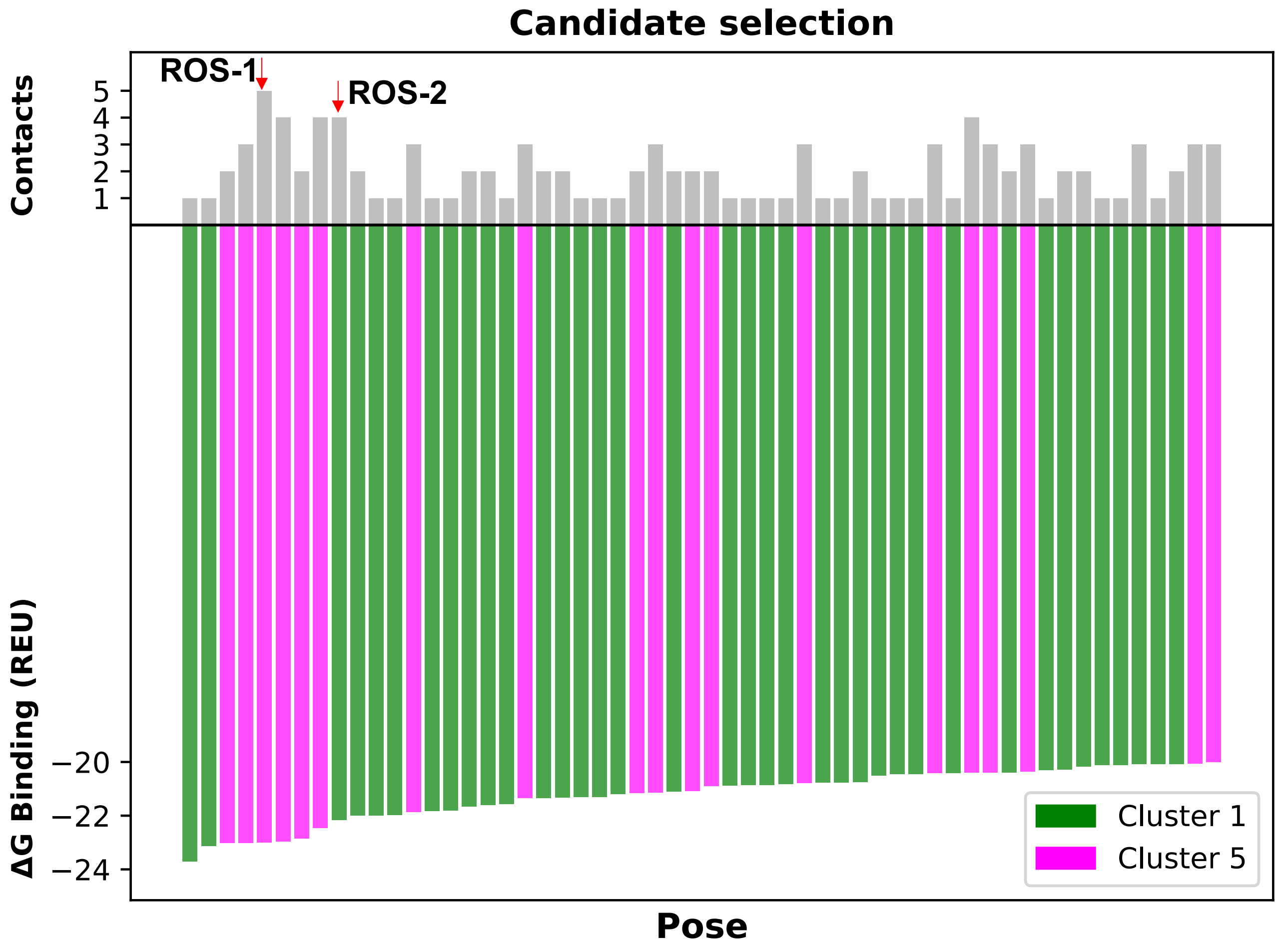


**Figure S23.** Application of the last stage elimination criteria for candidate pose selection for MD simulation noted as ROS-1 and ROS-2.

In the final stage of structure elimination, binding modes from clusters were selected based on the following criteria: $\Delta G$ Binding < -20 REU and important contacts > 1

As clusters 1 and 4 did not show more than one important contact they were eliminated and discarded from further analysis. Subsequently, poses from clusters 1 and 5 were ranked based on the lowest $\Delta G$ Binding energies and highest numbers of important contacts (Figure S23). The final selection included one structure from each cluster that presented both the lowest $\Delta G$ Binding and highest number of important contacts in their representative clusters. This resulted in the selection of ROS- 1 and ROS-2 binding modes from the parent clusters 5 and 1 respectively (Figure S23, Figure S2). ROS-1 displayed a total of 5 important contacts and $\Delta G$ Binding of –23.012 REU and ROS-2 displayed 4 important contacts and $\Delta G$ Binding of -22.159 REU (Figure S23).

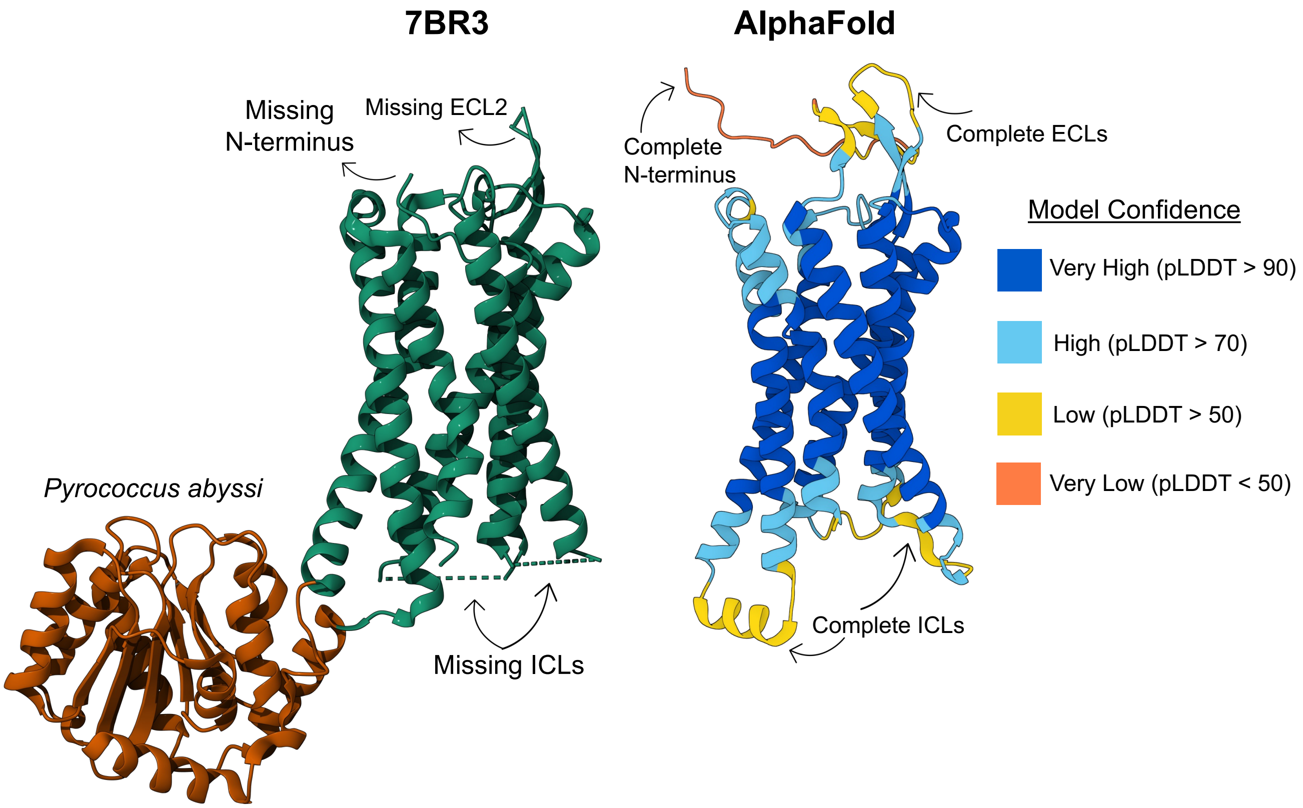


**Figure S24.** The crystal structure of the GnRH1R with PDBID:7BR3 on the left, in comparison with the AlphaFold predicted model of the wild-type 7BR3 on the right. The AlphaFold model includes the complete structure of 7BR3 where all the missing sequences were modelled and the Pyrococcus abyssi moiety is removed. The confidence level of the predicted model is depicted on the right with the confidence score indicator: pLDDT.

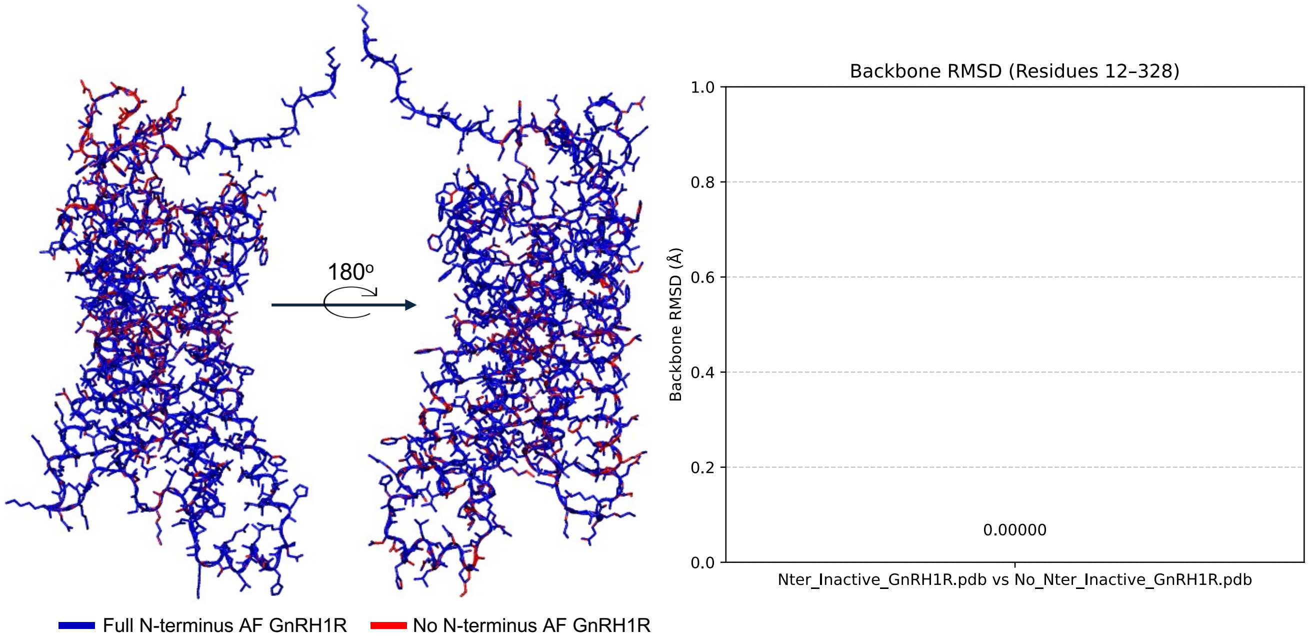


**Figure S25.** RMSD comparison of the Inactive GnRH1R predicted by AlphaFold before and after omission of the N-terminus for docking. RMSD values of zero indicate that the receptor was not influenced after the removal of the N-terminus.

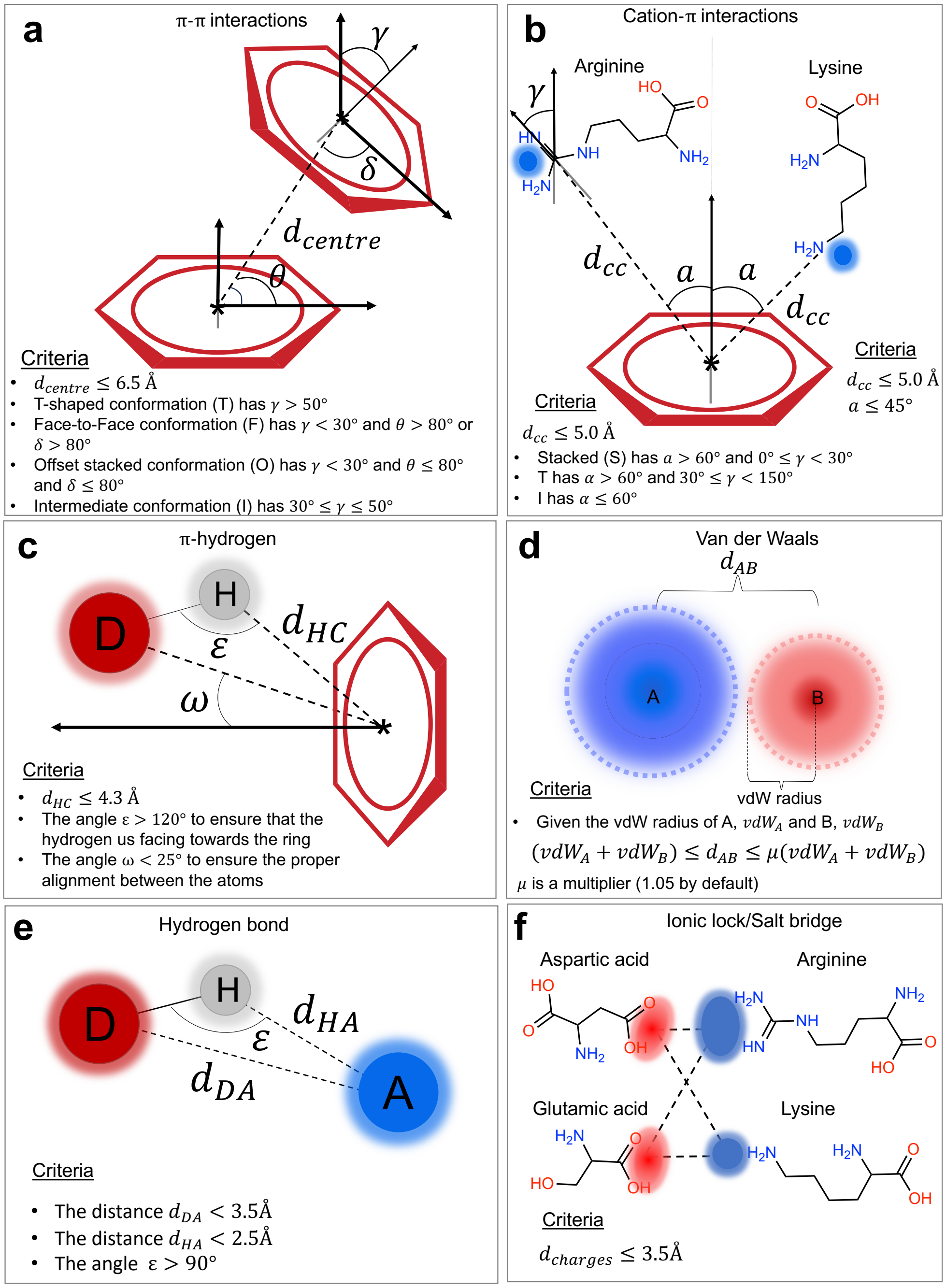


**Figure S26.** Examples of interactions calculated using RING: a) $\pi$-$\pi$ interactions, b) Cation-$\pi$ interactions, c) $\pi$-hydrogen interactions, d) van der Waals (vdW) interactions, e) hydrogen bonds, and f) Ionic locks (Salt Bridges). In the schematics, letter D represents electromagnetic donor atoms (nitrogen or oxygen), while letter A represents electromagnetic acceptor atoms.

## References


1. Bender, B. J. *et al.* A practical guide to large-scale docking. *Nat. Protoc.* **16**, 4799–4832, DOI: 10.1038/s41596-021-00597-z (2021).
